# Ideological biases in social sharing of online information about climate change

**DOI:** 10.1371/journal.pone.0250656

**Published:** 2021-04-23

**Authors:** Tristan J. B. Cann, Iain S. Weaver, Hywel T. P. Williams

**Affiliations:** Department of Computer Science, University of Exeter, Exeter, Devon, United Kingdom; West Pomeranian University of Technology, POLAND

## Abstract

Exposure to media content is an important component of opinion formation around climate change. Online social media such as Twitter, the focus of this study, provide an avenue to study public engagement and digital media dissemination related to climate change. Sharing a link to an online article is an indicator of media engagement. Aggregated link-sharing forms a network structure which maps collective media engagement by the user population. Here we construct bipartite networks linking Twitter users to the web pages they shared, using a dataset of approximately 5.3 million English-language tweets by almost 2 million users during an eventful seven-week period centred on the announcement of the US withdrawal from the Paris Agreement on climate change. Community detection indicates that the observed information-sharing network can be partitioned into two weakly connected components, representing subsets of articles shared by a group of users. We characterise these partitions through analysis of web domains and text content from shared articles, finding them to be broadly described as a left-wing/environmentalist group and a right-wing/climate sceptic group. Correlation analysis shows a striking positive association between left/right political ideology and environmentalist/sceptic climate ideology respectively. Looking at information-sharing over time, there is considerable turnover in the engaged user population and the articles that are shared, but the web domain sources and polarised network structure are relatively persistent. This study provides evidence that online sharing of news media content related to climate change is both polarised and politicised, with implications for opinion dynamics and public debate around this important societal challenge.

## Introduction

In spite of scientific consensus on the causes and primary effects of climate change, it remains a controversial topic in public and political discourse. Surveys have long shown substantial variation in public beliefs around climate change (for example [1, 2]) and the level of polarisation between individuals supporting and opposing action to mitigate anthropogenic climate change has been growing [3]. Media coverage of climate science and the frames used to present the information can have an important impact on public perceptions and willingness to take action [4], present different motivations and calls for action [5] and influence the accuracy and longevity of reproduced messages [6]. Recent work has shown that media effects vary depending on existing political biases [7]. Understanding the media landscape around climate change is of key importance in mapping public engagement with the issue and support for political actions to confront it.

Assessing which people are exposed to what information is fundamental to any study of the effects of media on public understanding and opinion. The disruptive impact of online media has transformed the media environment, radically altering the diversity of content people encounter as well as the exposure process itself. Individuals are faced with a wide range of media options (both social and traditional), new patterns of exposure (selected by the end user or driven by their social network) and increased production of user-generated content [8]. Previous work in this area has focused on the effects of incidental exposure on media awareness (e.g. [9]) and the diversity presented by online recommender systems (e.g. [10]). Such efforts are hampered by the diversity of online platforms, the rapid pace of their development and the obfuscation of the algorithmic processes they follow, and as a result no universal understanding of exposure effects is possible. Whether an individual is consuming the news online from a legacy media organisation, or producing and consuming information on social media, the fundamental dynamic of communication exposure and influence is that of network formation [11], based on creation of new relationships between users and media content by a variety of means (such as web-browsing and social information-sharing). Online media exposure creates a network that links sources and consumers of content (nodes) via their interactions (edges), requiring a network perspective for its proper understanding.

This study aims to describe patterns of sharing online media content about climate change. While a complete record of users’ exposure to digital content is only possible via accurate tracking of web browsing histories, media engagement can be inferred from the content users share on social media. Sharing a web article requires action by the user, and causes it to appear on the social media feeds of friends and followers, as well as contributing to aggregate trends, often advertised by social media platforms. This is used to indicate a significantly higher level of engagement than exposure. Social sharing of content instantiates a promotion mechanism, increasing the visibility of any content across a user’s social network and likely indicating that the sharer agrees with, or approves of, the content. These factors, along with the increasing use of social media as a source of news [12], mean that study of digital media sharing can provide insights into how information is propagated online, including important contemporary issues like climate change. In particular, Weaver et al. [13] shows that network analysis of such propagation patterns can reveal meaningful social structures of news engagement and consumption around political events.

Here we operationalise our study of online information-sharing around climate change by examining link-sharing on Twitter. User posts (tweets) referencing climate change and containing links to web content (URLs, which are often rendered into news blurbs or images in the Twitter client) were collected from the Twitter social media platform via its public API. This dataset was used to construct bipartite networks linking users to the digital media they shared. Analysis of network topology finds strong community structure, supplemented by comparison of source domains and textual content of articles shared within each community. Exploration of detected communities identifies strong ideological polarisation within the news-sharing network where users are segregated by divergent opinions, rather than an ongoing process, an alternative definition which is important in other contexts [14]. Overall the results indicate highly polarised and politicised engagement with web content around climate change, with largely segregated and ideologically biased communities receiving information from different media sources. We demonstrate that the observed correlation between political views and climate change beliefs (e.g. [3]) extends to online information sources and their shared readerships. For the first time, we track network structure over a 7-week period, including a disruptive media event (the announcement of the withdrawal of the USA from the Paris Agreement on climate change), to show the persistence of the observed polarisation and politicisation of media sharing related to climate change over time and with varying background levels of public interest.

Social media data has been used to study several aspects of public opinion around climate change, including attitudes towards and engagement with climate change mitigation strategies [15], media framing of the leaked “Climategate” emails on YouTube [5], and the spreading of calls for collective action at the COP15 conference [16]. Networks are an intuitive representation, and come with a host of analytical tools to understand the shape of online discussions around climate change; for example Elgesem et al. [17] explore the network of hyperlinks between blogs, while Williams et al. [18] study the structure of follower, retweet and mention networks on Twitter. In both of these studies, user communities manifest as densely interconnected clusters with similar characteristics. These communities are highly polarised, such that each community is well described by a single viewpoint, with few moderate voices. Similar patterns have also been observed for online political discourse (e.g. [19–24]). This pattern of opinion polarisation and segregation has important implications for opinion change and the likelihood of global consensus [25]. Polarisation in online social media is most frequently studied in the political sphere, especially for two-party political systems with an ideological split along a left-right axis. However, the phenomenon also extends to the competing opinions around climate change, which are often simplified as a debate between environmentalists (supporting the scientific consensus and promoting action) and sceptics (doubting or opposing the consensus and need for action), notwithstanding the diversity of viewpoints and representations of this complex issue [26]. These previous network-based studies generally treat datasets as single snapshots, along with the implicit assumption that the phenomena under study varies slowly. The intervals studied range from months to years, but by choosing such a timescale it is possible to overlook the changes that social networks can experience in shorter periods.

The pattern of polarisation in both political and climate change contexts is often associated with the existence of echo chambers in the social media ecosystem, whereby users choose to associate with people and news-media sources which conform to and reinforce their existing beliefs [18]. Echo chambers have been proposed to contribute to the spreading of misinformation [27], political networks of environmental actors [28], exposure to political information on social media [21], and online content around climate change (e.g. [17, 18, 29]). In this work we focus on the structural phenomenon of echo chambers but other scholars have looked at how they are linked to psychological processes such as confirmation bias (e.g. [30]).

Previous studies of information-sharing around climate change have focused on the prominence of different sources. Newman [31] analysed the tweets and information sources shared alongside the release of the IPCC AR5 WG1 report, finding a focus on the public engagement with science, and a dominance of mainstream media sources. Segerberg and Bennett [16] examined the breakdown of different link sources used alongside calls to collective action at the COP15 conference. Kirilenko and Stepchenkova [32] studied the URLs shared on Twitter over the course of one year in five different languages, finding that by country, the US dominated total tweet counts, and a mix of traditional media, activist and sceptic sites were shared.

Polarisation is a common observation in the climate change debate. Notable studies such as Dunlap et al. [3] have shown that the polarisation effect in climate change opinion has grown between 1997 and 2016. An impact of polarisation can be observed in the frames used to discuss climate change, such as by Jang and Hart [33], who analysed the themes present in Twitter’s climate change debate across two years. They found differences in the terminology used by opposing groups, with Republican-leaning states in the US using *global warming* in preference to *climate change*, and often using hoax frames to cast doubt on the scientific consensus. O’Neill et al. [34] found that this trend extended to the media coverage of the publication of the IPCC AR5 working group reports. By studying the frames used in newspaper and television broadcasts, they found clear preferences for different frames amongst the various news organisations. Some work has looked at countering the growing levels of polarisation, including Zhang et al. [7] who study the effect of clarifying messages on accuracy over the perceived levels of consensus among climate scientists. Among their experimental group, exposure to the clarifying message lead to more uniform accuracy around the scientific consensus through greater impact in the areas of lower baseline belief.

The effects of polarisation do not always manifest equally on each end of the opinion spectrum. Schuldt et al. [35] compared the usage of the terms *climate change* and *global warming* across the websites of a series of think tanks. Right-wing think tanks were more likely to use *global warming*, with the opposite trend observed in left-wing think tanks. These findings of content differences from polarised sources extends beyond the climate change debate. Further analysis of climate sceptic organisations and their funding sources by Farrell [36] found shared sources of funding across many of them. Beyond the topic of climate change, Freelon et al. [37] present an overview of how the different online activism strategies of left- and right-wing groups manifest different types of content and audiences. They highlight a key difference in the perceived strategies of the different groups. Left-wing groups target “hashtag activism” leading to social promotion of movements whereas right-wing groups engage much more readily with sympathetic media organisations to promote their messages and goals. Freelon et al. also recognise a similar trend in group coherence where right-wing groups are tighter when compared to left-wing groups formed from a loose coalition of multiple issue-led groups. Considering this coordinated funding of multiple groups and engagement with media organisations, it is to be expected that climate sceptic messaging is likely to be more consistent than competing environmentalist information.

Along with climate change, studies of information-sharing on social media have embraced a diversity of different topics; Starbird [38] studied the spread of misinformation around mass shooting events in the United States, finding a cluster of alternative news sites separate from mainstream media sources. Arif et al. [39] and Del Vicario et al. [27] study the spreading behaviour of rumours and misinformation on Twitter and Facebook respectively and classify observed trends. Schmidt et al. [40] analyse a network of news-related pages on Facebook, where two pages are connected if they have posts that are liked or commented on by the same user. Their cluster analysis reveals a highly polarised structure, as seen in a number of other contexts (e.g. [19]). Williams et al. [41] also study the community structure of political news-sharing via Twitter, finding communities characterised by both geographical and political factors. Weaver et al. [13] examined information-sharing on Twitter during the UK General Election in 2015, showing strong community structure explained by ideological, geographical and topical preferences. Each of these studies should be considered in the context of the typical sharer and the information they are exposed to. Not all users on social media are exposed to the same information, and typically they are exposed to information in which they have already shown an interest through their own decisions of which users to follow, along with algorithmic filtering effects. Sharing information requires action on the user’s part and as such those who choose to share are likely part of a more highly invested subset of the users exposed to a certain piece of information.

The rest of the paper proceeds as follows. Section Methods details the method of data collection and preparation, along with the techniques used to construct and analyse the information-sharing networks. Our main results follow in Section Results, including network analysis and characterisation of community structures, and finally Section Discussion provides a thorough discussion of the main findings of this study and places them in the wider context of media effects on the climate change debate. Additional data and visualisations can be found in the S1 File.

## Methods

### Tweet collection and pre-processing

This analysis uses seven weeks of Twitter data collected from the Streaming API [42]. Tweets containing the strings *climate change* or *global warming* were collected between 2017-05-10 and 2017-06-27, giving an initial dataset of 5, 320, 400 tweets by 1, 975, 593 users. Inspection suggests that most of the content comes from the US and the UK.

The collection period captures a key event in the unfolding climate change narrative, the announcement on 1st June 2017 by then-US President Donald Trump that the USA would withdraw from the Paris Agreement on climate change mitigation. This event caused a large spike of activity on Twitter around the topic of climate change illustrated by S1 Fig in S1 File. To study this event in the context of a longer period of “normal” activity, the collection spans seven weeks centred on the week of greatest activity. We separate the dataset into seven one-week intervals to account for the potential weekly periodicity in social media usage and gives sufficient sampling density for robust network analyses.

This study focuses on the digital media engagement and sharing behaviour of Twitter users discussing climate change. To capture this behaviour, the dataset is filtered to remove all retweets (where a user reposts an original tweet) and quotes (where a user reposts an original tweet with their own commentary prepended). The purpose of this filter is to focus on original tweets, which are assumed to be the strongest available measure of user engagement with content; the low effort cost of retweeting and quoting means that user engagement cannot be inferred as clearly from these tweet types. The remaining tweets are further filtered to retain only those which contain an embedded link (URL) to web content. These links are the digital media items shared by the tweet author, including news articles, blog posts, videos and other content. Such tweets can be composed by manual insertion of a link or clicking the ‘share’ button often presented alongside online news content. Application of all the filters leaves a dataset of 592, 830 tweets containing URLs by 195, 134 users, broken down over the 7-week study period in Table 1. Most notably, the centre point of the dataset in week 4 includes the US intention to withdraw from the Paris Agreement, producing a dramatic surge in Twitter activity.

**Table 1:** Weekly tweets and unique users in our filtered dataset. Week 4, in which the Paris Agreement announcement was made, includes many more tweets than any other week, accounting for 35% of all the tweets studied.

Week	Original Tweets	Unique users
1	52, 737	27, 499
2	60, 922	31, 769
3	78, 353	38, 683
4	209, 637	96, 506
5	67, 234	36, 620
6	65, 248	34, 625
7	58, 699	28, 536

### URL validation

Every URL found in the tweet dataset was validated in December 2017 to ensure that it was a working link to an identifiable item of online content. This validation step was necessary for a number of reasons. Firstly, each URL must be accessible to allow subsequent analysis of content. Secondly, typographical errors by tweet authors are sometimes incorrectly interpreted by the Twitter API as URLs, such as periods followed by alphanumeric characters (*hello.world*). Thirdly, the validation process handles shortened URLs, which represent the majority of URLs in tweets. Many Twitter users make use of third-party URL shorteners, services which create a redirect to a given long URL from a shorter version that can then be used to reduce the characters needed to embed URL in a tweet (this is unnecessary since Twitter includes its own shortening service that reduces any URL to 23 characters). URL shorteners are also sometimes used to conceal the target URL from anyone who may potentially click on it. These short links must be traced to their destination both to ensure they point ultimately to a valid resource, and also to be treated alongside other links to the same destination.

Each URL is resolved individually, marking as valid only those that return HTTP status code 2XX immediately or through a small number of permanent redirects (301 → 301 → … → 2XX status codes). The 2XX status codes indicate various successful outcomes to an HTTP request.

Many valid URLs included modifiers inserted for tracking and metadata purposes, without altering the destination page. This can lead to many URLs pointing to the same resource, so to minimise the possibility of considering these as distinct URLs, all schemes and queries are removed from the resolved URLs. A small number of domains were adversely affected by this. YouTube and other Google services were the most prominent, largely due to the structure of their links. As a result, our process does not distinguish between different YouTube videos within the networks.

The validation process results in a mapping between each raw URL (as given in a tweet) and the validated final destination after any acceptable redirect paths. Any tweet containing at least one unvalidated URL is removed from the dataset. Finally, all users who tweeted more than 50 times within a given week are removed; this threshold is found to be a sensible limit to mitigate the impact of automated accounts, especially news aggregator accounts. Across the seven weeks 271 unique accounts were affected, leading to the removal of 66, 892 tweets (approximately 1% of the total dataset). This left 245, 446 tweets by 113, 154 users sharing 54, 462 distinct, validated, URLs across all of the seven one-week windows.

### Network construction

**Fig 1:**
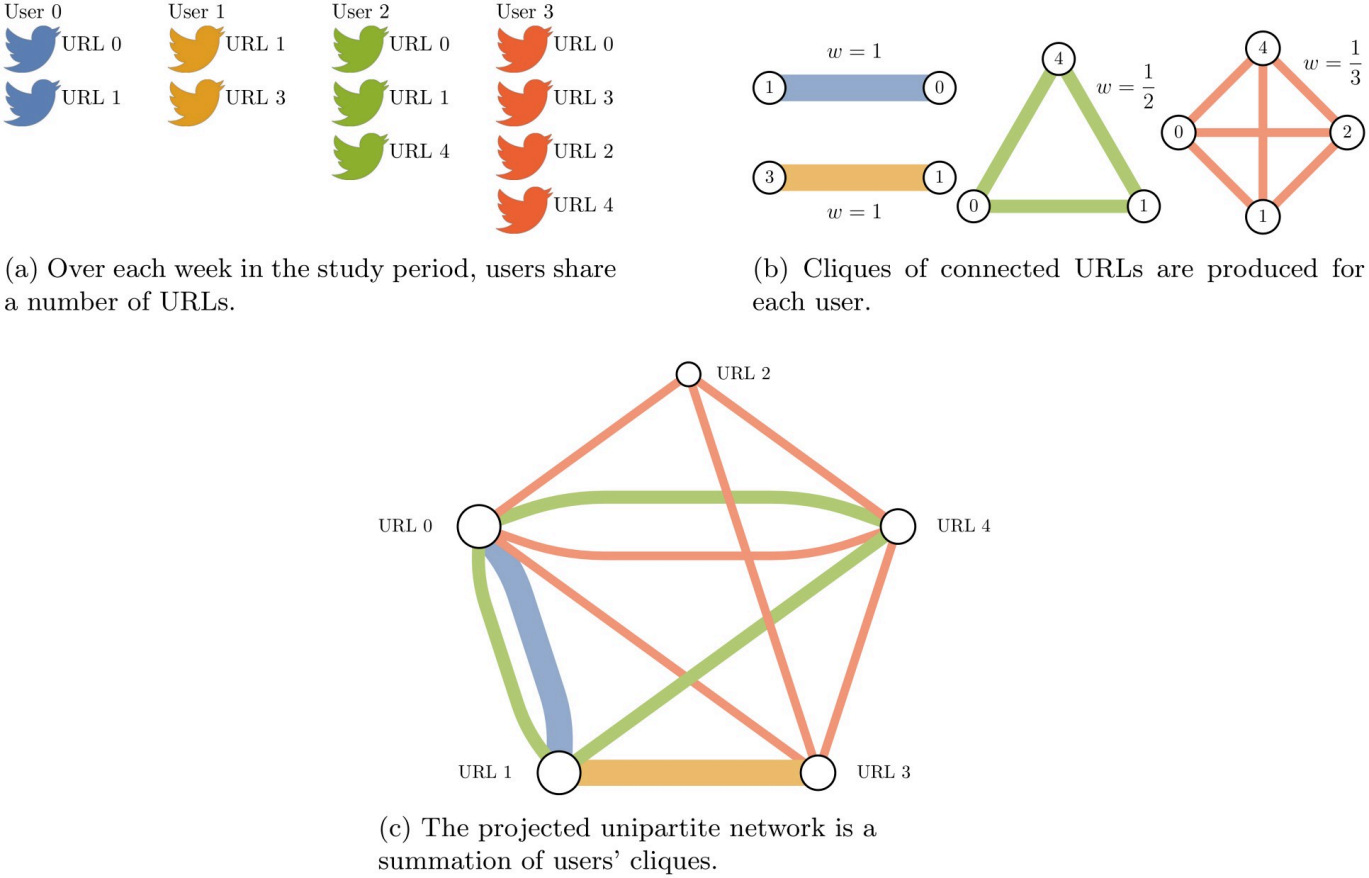
Schematic diagram of the bipartite network construction and unipartite projection. Each user is connected to the URLs they share in the study week. The unipartite projection creates edges between two URLs whenever they are shared by the same person. Multiple edges in the projection indicate that multiple users have shared the pair of URLs, and this information is tracked by edge weights. A user’s edge contribution to the projection increases quadratically with the number of URLs they share, potentially leading to a dominance of highly active users in the unipartite projection, for example User 3 (red) in the projection; this is handled by a hyperbolic weighting scheme (see Section Network construction).

Bipartite networks, containing two node classes representing users and URLs, are constructed by creating an edge *i* → *j* whenever user *i* shares URL *j*, illustrated by Fig 1. Multiple shares of URL *j* by user *i* increment the edge weight so that the final bipartite edge weight *w*(*i*, *j*) represents the number of times user *i* shared URL *j*.

Next a unipartite projection is produced from the bipartite network, whereby a network of only URL nodes is created, where edges encode the number of users that shared the connected pair of URLs. The network construction process is illustrated in Fig 1. The URLs shared by a single user (Fig 1a) form a fully connected clique of nodes representing the sharing pattern for that user (Fig 1b). The whole unipartite network is then constructed by composing the cliques for all users (Fig 1c). The projection allows the use of efficient unipartite network analysis algorithms and focuses the analysis on the relationships between URLs. The unipartite network of URLs represents the collective pattern of sharing online content of the targeted Twitter user population. Statistics for the seven one-week networks created are given in S1 Table in S1 File.

We restrict our analyses to the giant component to avoid the issue of network fragmentation and limit its impact on community detection. In all cases the giant component consists of around two thirds of all URL nodes in the network. One important design decision made in this process is how user shares are encoded into unipartite edge weight; different weighting schemes significantly impact the performance of community detection algorithms. User contributions to unipartite edge weights are scaled by the factor 1/(*k*_*i*_ − 1), where *k*_*i*_ is the total number of article shares by user *i* (including repeated links), such that the total unipartite edge weight contribution by each user is *k*_*i*_/2 (as proposed in Newman [43]). This hyperbolic weighting scheme was found to allow robust recovery of community structures after projection [44]. Without this weighting factor, a user’s edge weight contribution to the projected network is quadratic in the number of URLs shared, since each user creates *k*_*i*_(*k*_*i*_ − 1)/2 edges in the projection, causing users who share many articles to quickly dominate the network and subsequent analysis.

Computing the projection requires knowledge of the biadjacency matrix *B* of the giant component, where the rows represent users and the columns represent URLs; and a diagonal matrix *D* such that *D*_*ii*_ = 1/(*k*_*i*_ − 1). Given these matrices, the adjacency matrix for the weighted user projection *P* = *B*^*T*^ *DB* and hence the weight of an edge between URLs *i* and *j* in the projection

$$\begin{array}{c} w\left(i,j\right)=\sum_{u\in \text{Users}}\frac{w(u,i)w(u,j)}{deg(u)-1}. \end{array}$$ (1)

### Community detection

Community detection was used to find clusters of densely interconnected nodes in the network, which in this context represent sets of URLs which were seen by a group of similar Twitter users. Community detection [45] is a means of algorithmically identifying such clusters in a given unipartite network. This study used a greedy algorithm proposed by Clauset et al. [46] that partitions nodes into communities such that the modularity of the partition is optimised; modularity measures the proportion of edges within communities relative to the proportion of edges between communities. If a community assignment is significantly better than random, a modularity score between 0.3 and 0.7 is typically observed [45]. Modularity scores for each network are given in S1 Table in S1 File.

### Content analysis

Page content was collected for each validated URL using Diffbot [47], an online service for extracting the constituent parts of an HTML document and presenting them in an easily analysed format. In some cases, Diffbot only identifies the title and cannot automatically detect the content of a webpage. To mitigate the impact of this, the title is used as the page content for such URLs. This substitution affected less than 5% of the URLs in each week, except Week 4 which required substitution in 5.9% of the URLs. Additionally, a small number of domains were incompatible with the Diffbot API. This accounts for around 4% of all URLs in the giant components of any week and mostly arise due to page formatting or timeout issues.

Online content was analysed quantitatively by calculating term frequency-inverse document frequency (TF-IDF) scores, treating each URL as a document and the set of all URLs in the whole network as the corpus. TF-IDF analyses aim to identify distinctive or important tokens (usually words) in a document, based on their frequency in the document relative to their frequency across the corpus. Three kinds of token were studied in this way, in three separate analyses: web domains, page content unigrams, and page content bigrams. Web domains were extracted for each URL to permit analysis of the sources of content shared on Twitter. Words and bigrams (two-word phrases) were extracted from the web pages associated with each URL to allow large-scale analysis of the topics within the content. Before calculating the TF-IDF vectors for page content, the Snowball stemmer [48] was applied to each content token. Each stemmed token is mapped back to the most common token that maps to it for ease of reading, e.g. if fisher, fished and fishing all appear once and fishes appears twice then the final representation of these tokens is fishes.

TF-IDF score vectors for each URL are calculated and represent the frequency of tokens in a document relative to their frequency across the corpus. For token *t* in document *d* we have

$$\begin{array}{c} \text{TF-IDF}\left(t,d\right)=tf\left(t,d\right)(\text{log}(\frac{1+n}{1+df(t)})+1), \end{array}$$ (2)

where *n* is the number of documents in the corpus, *tf*(*t*, *d*) counts the frequency of *t* in *d* and *df*(*t*) counts the number of documents in the corpus which contain *t*. To characterise each community found in the sharing network, the TF-IDF vectors for each URL in a community were summed to obtain aggregate scores. In these community-level comparisons, a high TF-IDF score for a community means that the given token appears more frequently in the URLs in this community, when compared to other communities. In each case, tokens which occur in fewer than 50 URLs, or more than 50% of all URLs in a given week are rejected, such that very common or very rare tokens are omitted. We also removed a set of common stopwords including *trump*, *paris* and *agreement*. In principle, *n*-grams can be studied with any value of *n*, but beyond *n* = 2 token frequency is generally too low to be useful.

### Ideological coding of source domains

To examine ideological positioning of popular source domains along axes of political leaning (from left/progressive to right/conservative) and climate scepticism (from environmentalist to sceptic), 62 of the 75 most commonly shared source domains across the entire study period were manually coded for ideological bias (listed in S4 Table in S1 File). Ideology expressed in articles from each domain was graded on a three-point scale for political opinion (Left-Neutral-Right) and climate change opinion (Environmentalist-Neutral-Sceptic), with an additional null rating (Unclear) added to both scales for cases where no clear ideology was seen.

A team of six human coders used this scale to independently score text extracts of articles/content from the most commonly shared domains. For the 75 domains with the most shares across the seven-week period, we sampled the page content for five most shared articles (using Diffbot to extract clean text, as described above). If fewer than five articles from a popular domain were available in the dataset, then all were included. In some cases, the page formats were incompatible with the Diffbot API, returning no content for 10 domains (see S4 Table in S1 File for the excluded domains). We additionally excluded the social media sites *Twitter*, *Wordpress* and *Reddit*; these do not have editorial control and therefore lack a unified ideological position. This left a set of 62 domains to be coded. Each extract consisted of up to three complete paragraphs (of at least 30 words) from the linked web page text. To limit any subjectivity arising from the coders’ personal perspectives, each extract was anonymised (i.e. source domain and author information were removed) when presented for coding.

Coders were provided with the following definitions to help make their assessments:

- *Left*: A left-wing stance can be characterised by the promotion of state benefits and services, public investment in and regulation of private businesses, increased taxation of corporations and high earners, and support for workers and trade unions.
- *Right*: A right-wing stance can be characterised by promoting low taxation and minimising the interference of government in personal and business lives. Public investment is minimised, in favour of allowing market forces to control growth and provision of services.
- *Environmentalist*: An environmentalist stance supports the scientific consensus on anthropogenic climate change and promotes immediate action by governments and individuals to mitigate the future impacts.
- *Sceptic*: A climate sceptic stance opposes the scientific consensus on anthropogenic climate change. Such opposition varies from questioning the existence or causes of climate change, to opposing efforts to mitigate its impacts.

Each coder assigned a score to each article extract independently. That is, six coders generated up to five scores for each of 62 domains (in practice, this amounted to 1698 scores out of a maximum of 1860, with each domain receiving up to 30 scores). Each article score is an integer denoting the level of ideological bias the coder observes in that article (-1/0/1 for left/neutral/right or environmentalist/neutral/sceptic, in order, with unclear scores ignored; see S3 Table in S1 File). The article scores assigned by each coder were then averaged for each domain to determine an overall domain score for that coder. Each domain score is a real-valued number in the range [−1, 1]; there are 6 scores for each domain, one from each coder.

Since the assignment of ideological bias is somewhat subjective, we performed an adjustment to the domain scores assigned by each coder. This adjustment normalised each domain score by subtracting the mean domain score for that coder across all 62 domains, then dividing by the standard deviation. This process represents domain scores as *z*-scores and normalised for subjective bias of individual coders. Finally, we average the normalised domain scores across all coders to find a single domain score. This process was applied for both political and climate change ideology. Since judgment of climate change ideology relies on some knowledge of climate change (e.g. the scientific consensus) the coders were recruited from the postgraduate research community in sciences at the University of Exeter.

**Fig 2:**
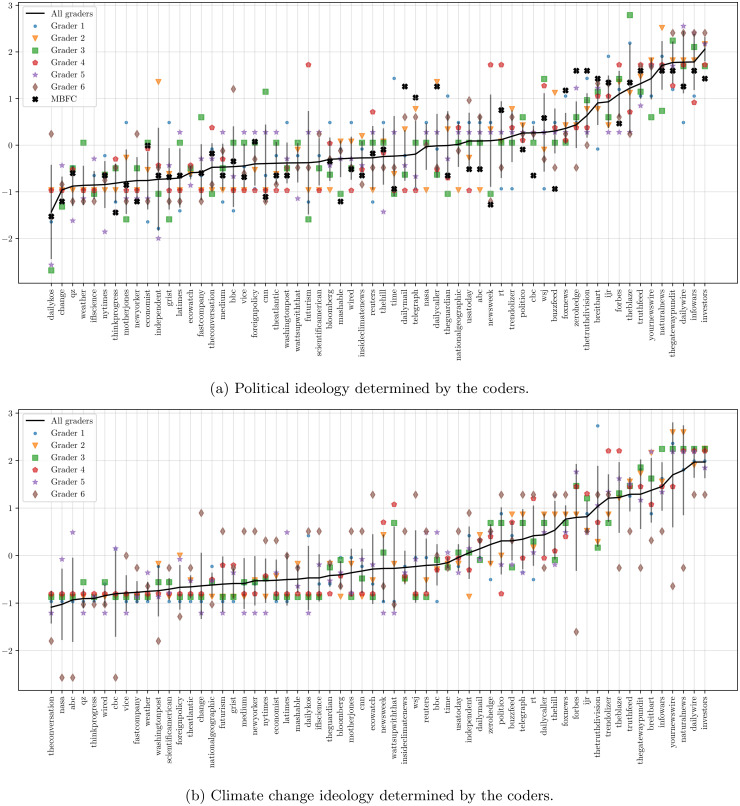
Average domain ideological positions assigned by each coder and the overall average across all coders. Values should be thought of as standard deviations from the mean. Vertical bars indicate ± one standard deviation across the coders.

Full results for the coding of each domain are presented in S2 Fig in S1 File. To test the reliability of our coding exercise we compare our grades to the domain assessments by Media Bias/Fact Check [49] (MBFC), a fact-checking and media bias site. We translate the MBFC ratings to our own scale and apply the same *z*-score normalisation, then calculated the correlation of the political bias scores by our coders against those from MBFC for those domains which have been rated by MBFC. We find strong positive correlation (Pearson’s *r* = 0.8, *p* < 10^−6^, *n* = 49) demonstrating that the political coding process is consistent with the expert assessments provide by MBFC. No equivalent external assessments are available for climate change ideology, but the robust performance of our coding process for political bias gives confidence in the methodology. See Fig 2a and 2b for the average results across domains and coders for political and climate change bias respectively.

### Comparisons over time

To measure the change in patterns of sharing climate media over time, the pairwise similarity of the sets of users, articles (URLs) and source domains was calculated for the seven weeks in the study period. An asymmetric similarity measure is defined to give an indication of persistence between weeks and identify influxes of new participants or content. This expression compares weeks *A* and *B* by the fraction of the users, *u*, links, *l*, or domains, *d*, that appeared in week *A* that also appeared in week *B*. In order to account for the repeated usage common in online social networks, a measure of how many times a user, URL or domain appears in each of the two weeks is included by using multisets for *u*, *l* and *d*.

$$\begin{array}{c} S_{A,B}^{u}=\frac{|u_{A}\cap u_{B}|}{|u_{A}|},\;S_{A,B}^{l}=\frac{|l_{A}\cap l_{B}|}{|l_{A}|},\;S_{A,B}^{d}=\frac{|d_{A}\cap d_{B}|}{|d_{A}|}. \end{array}$$ (3)

Values of the similarity measure fall within the range [0, 1]. Values approaching 0 signify that very few of the users (respectively URLs, domains) in week *A* are also present in week *B*, whereas values approaching 1 signify that nearly all of the users (respectively URLs, domains) in week *A* are also present in week *B*. Note that by design *S*_*A*,*B*_ ≠ *S*_*B*,*A*_ for *A* ≠ *B* in general.

## Results

This section divides our results into three main findings. In the first part we focus on Week 4 of the study period, in which the US withdrawal from the Paris Agreement was announced, to demonstrate the broad ideological polarisation observed in the information-sharing networks. Secondly, we characterise the sub-communities that make up the network, showing that several linked left-wing/environmentalist communities co-exist with a single right-wing/sceptic community. Finally, we look at all seven weeks in the study period to explore how network structure and polarisation changes over time.

### Climate media sharing is polarised and politicised

**Fig 3:**
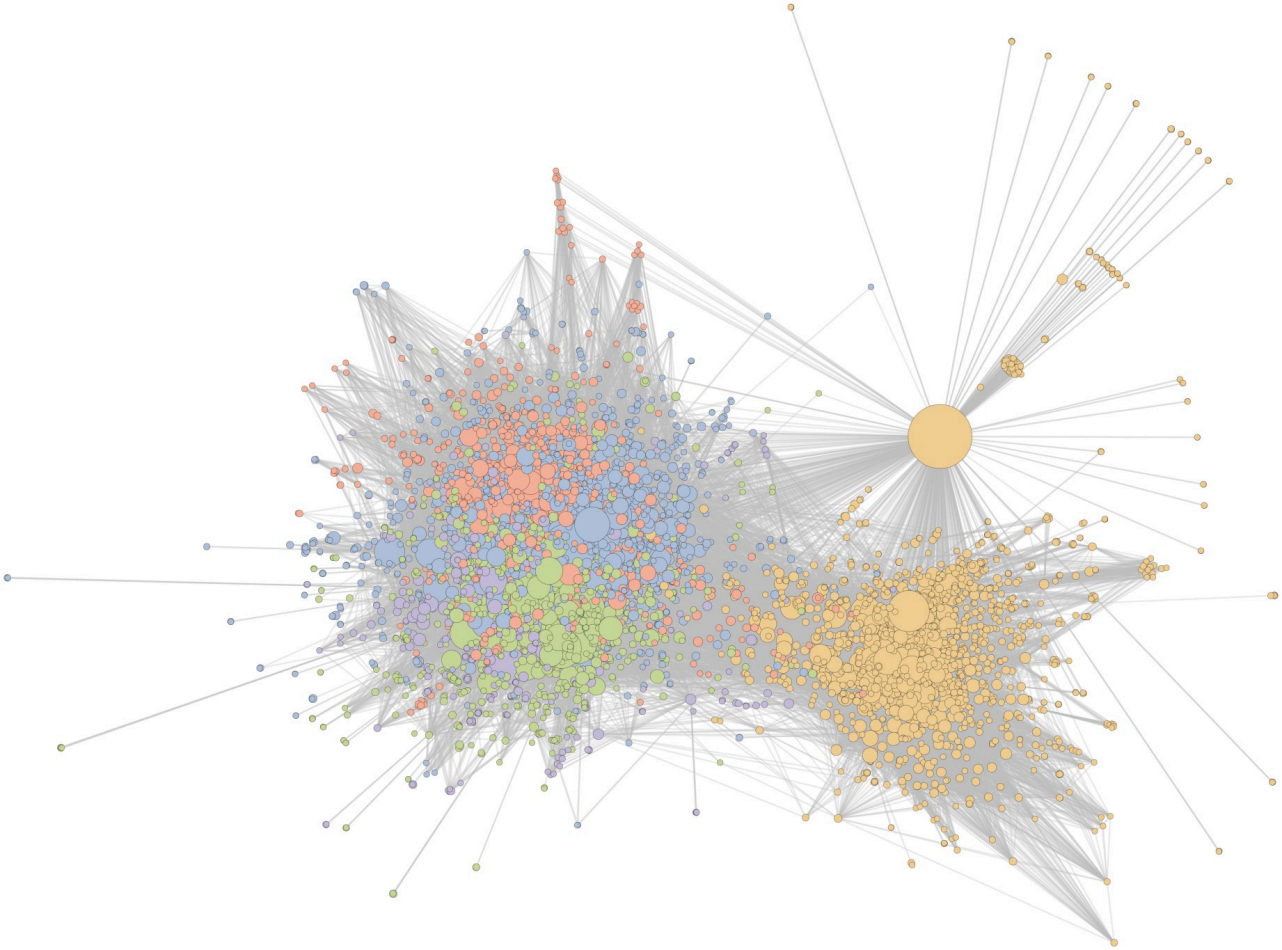
Information-sharing networks are polarised. Plot shows the URL co-sharing network for the week in which the US withdrawal from the Paris Agreement was announced (Week 4 of the study period). The five largest communities by total share count are displayed (67.69% of 7, 496 nodes). Communities 1 − 5 are coloured blue, yellow, green, red and purple respectively and node size is proportional to the square root of total share count. Node placement uses a force-directed algorithm [50] which groups densely connected nodes together; this layout highlights two large clusters, with four communities on the left and a single community on the right.

We begin by characterising the information-sharing network during the central week of the study period, Week 4, in which the US withdrawal from the Paris Agreement was announced. During this week, 7, 496 URLs were shared by 42, 113 Twitter users. After projection this produces a unipartite network of 7, 496 URLs connected by 107, 304 edges indicating which pairs of URLs were co-shared. Fig 3 shows the URL co-share network of the five largest communities by total share count. The layout is determined by the ForceAtlas2 algorithm [50], which groups densely connected nodes together; visual inspection shows a clear partition into two large clusters. Algorithmic community detection reveals further partitioning within the larger cluster of the network, illustrated by the different node colours. By considering the contrasting structures found by the community detection and network layout algorithms, there is evidence of multiple layers of structure within the network. Note that the large yellow node separated from the two main clusters is *youtube.com/watch*. This node is difficult to interpret as it aggregates many YouTube video links, seemingly from all sides of the debate. We cannot verify this with our text-based content analysis.

**Fig 4:**
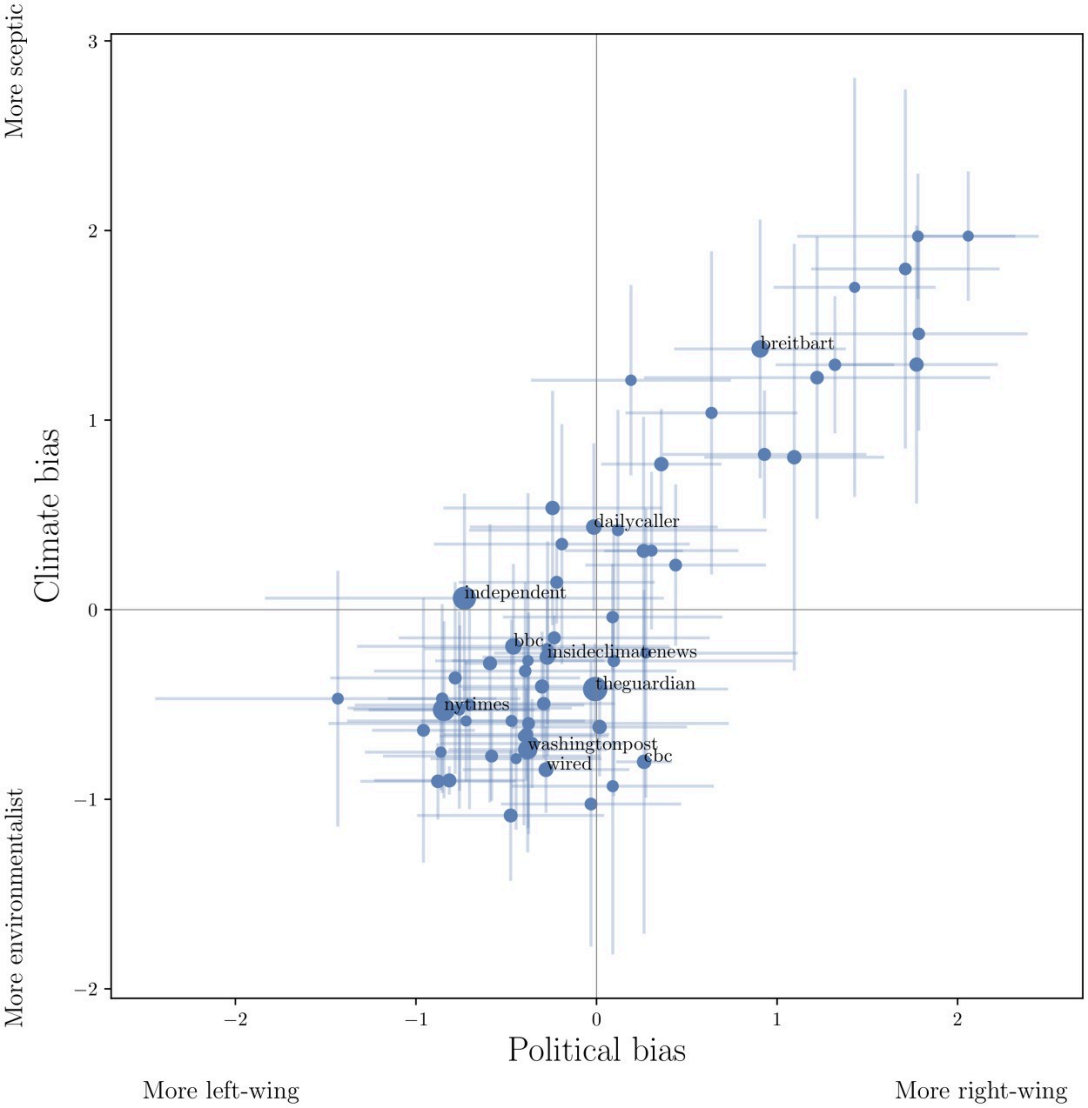
Climate media content is politicised. Mean political ideology (left-to-right) and climate opinion (environmentalist-to-sceptic) expressed in content from the 62 coded web domains over the six coders (see Ideological coding of source domains). Point size is proportional to the square root of total share count and lines indicate ± one standard deviation. Labels are shown for 10 most frequently shared domains in the coded list.

There is a strong correlation between opinions about climate change and political ideology expressed in shared content. Fig 4 plots the position of the 62 web domains on axes of climate bias and political bias, based on content of articles coded by the panel. Left-right political ideology and environmentalist-sceptic climate opinions are very strongly correlated (Pearson’s *r* = 0.86) and very few domains appear in the right-wing/environmentalist and left-wing/sceptic quadrants.

**Fig 5:**
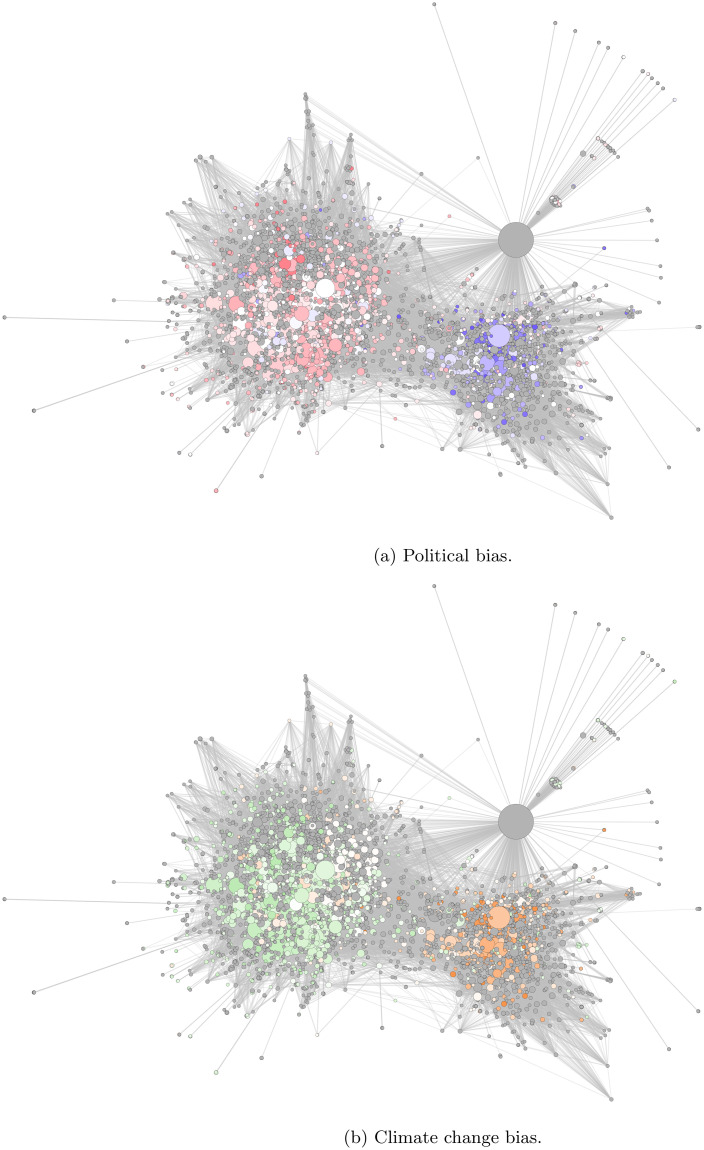
Network clusters are ideologically biased. The two large clusters within the URL co-sharing network for Week 4 shown with URLs coloured by: (a) the average political bias of their source domain; and (b) the average climate change bias of their source domain. Red denotes left-wing domains, blue denotes right-wing domains, green denotes environmentalist domains, orange denotes sceptic domains. White denotes any domain coded as neutral and domains not coded are in gray.

Mapping ideologies/opinions associated with web domains onto the information-sharing network structure shows an association between network position and viewpoint. Fig 5 shows the network diagram from Fig 3 with URL-node colours altered to show biases in the political/climate opinions expressed by their web domains. Fig 5a colours the nodes to highlight the left-right political bias. The left-hand cluster contains predominantly left-wing sites. The right-hand cluster has a high concentration of right-wing sites, but also has many uncoded sites. A similar pattern can be seen in Fig 5b, which colours nodes by climate change bias, with the left-hand cluster predominantly environmentalist and the right-hand cluster containing most of the sceptic domains. Taken together, these findings demonstrate significant strength of polarisation and politicisation in information-sharing about climate change on Twitter, with two large clusters of users and information sources, broadly characterised as a left-wing/environmentalist group and a right-wing/sceptic group.

### Characterisation of information-sharing communities

**Fig 6:**
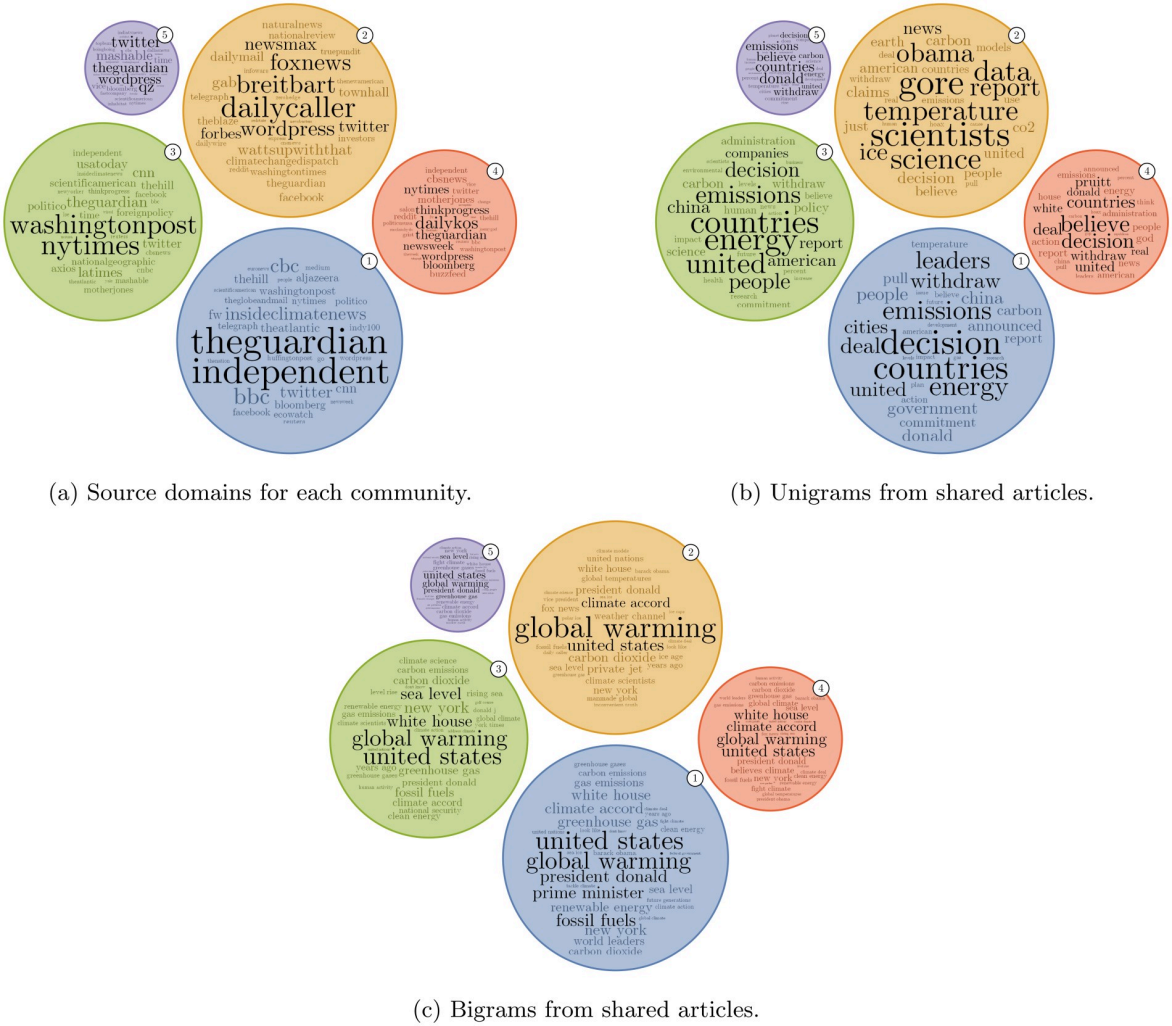
Source domains and content of media articles shared within the five largest communities in the information-sharing network. Tokens in each plot are weighted (using TF-IDF) to make distinctive tokens prominent. Circle size is scaled to indicate the total number of URL shares within the community. Terms coloured black are the highest weighted terms required to reach 15% of the total weight in a community. For visual clarity, each stemmed token is represented by the most common token that maps to it (or pair of tokens for bigrams).

The main source domains and indicative content of media articles shared within the five largest communities in Week 4 can be seen in Fig 6, in which the radii of the circles show the relative sizes of these major communities. Here Communities 1, 3, 4 and 5 are communities within the left-wing/environmentalist cluster in the information-sharing network, whereas Community 2 is the single community in the right-wing/sceptic cluster. Fig 6 uses a TF-IDF weighting scheme for each token, such that the prominent tokens are those that are characteristic of a particular community when compared to the network as a whole. Source domains are shown in Fig 6a and content is shown in Fig 6b (unigrams) and Fig 6c (bigrams), to allow a characterisation of the broad themes in each community in terms of geographic focus, political or climate science biases and key subjects of interest. Table 2 summarises these communities using the data from Fig 6.

**Table 2:** Characterisation of the five largest communities in Week 4.

Community 1	This is the largest community and is dominated by mainstream media outlets, such as *The Guardian* and *The Independent*, mostly from the UK. This community includes mainstream news reporting and discusses the Paris Agreement on climate change almost exclusively, focusing on the consequences of President Trump’s decision and any international responses.
Community 2	This community includes many right-wing sources, including alternative news sites such as *Breitbart* and *The Daily Caller*. Social media sites are also prominent in this community as *Facebook*, *Twitter*, and *Gab* all appear, suggesting that this group captures attempts to use Twitter to re-share content from other social platforms. Some established media outlets such as *The Daily Mail* and *Fox News* are present, but are less focused in this community than alternative news sites. This community also discusses the Paris Agreement decision made by President Trump, as well as certain aspects of climate science. This is the only community to focus on former US politicians *Obama* and *Gore* and the phrase *global warming* dominates the bigram cloud (Fig 6c).
Community 3	This community consists of many mainstream domains revealing a US focus. The most prominent domains here are established mainstream media sources such as the *Washington Post* and *New York Times*. As with Community 1, the Paris Agreement on climate change is a key topic of interest.
Community 4	This community includes a number of alternative and smaller news media domains with a mostly left-wing bias, such as *Daily Kos* and *Mother Jones*, amongst established mainstream news sources. The content here is similar to that of Community 1 and Community 3, but also references then US Environmental Protection Agency Administrator Scott Pruitt and Michigan congressman Tim Walberg for their comments around climate change.
Community 5	This community is comprised of a mix of social media, news and commentary sites. Again, the Paris Agreement decision is a focus, with additional framing around global consequences and opinion-pieces on the decision.

Looking at source domains (Fig 6a), different geographic and political biases can be inferred based on earlier analysis of domain ideology. Community 2 features predominantly right-wing sources, whereas Communities 1 & 3-5 contain content from left-wing sources. Terms such as *decision* and *withdraw* in each of the communities show that the Paris Agreement announcement is a major topic of conversation during this seven-day period, even spanning the ideological/geographic divisions illustrated by Fig 6a. The left-wing communities are reasonably similar to each other, while the right-wing community is unique in its mention of previous US political figures such as *Obama* and *Gore* and use of scientific terminology. The bigrams mostly confirm the findings in Fig 6b, but also highlight differences in terminology, e.g. greater prominence of *global warming* in right-wing Community 2. Fig 6 and Table 2 demonstrate that there is variation in the geographic scope of the communities. The most apparent contrast exists between Community 1, which heavily features UK news sources, and Communities 2 and 3, which include mostly US sources. The distribution of words and bigrams in Fig 6b and 6c show some topical differences between the four communities in the left-wing cluster. Communities 3 and 4 include more terms related to the consequences of the decision for the *American* people whereas Community 1 is mainly concerned with the international political ramifications. These findings suggest greater focus and coherence amongst the right-wing and climate-sceptic frames and support the findings of [37], with greater fragmentation amongst the left-wing and environmentalist frames; this may partly reflect the larger size of the left-wing/environmentalist cluster in the information-sharing network, which permits greater internal differentiation.

### Consistency of network structure over time

**Fig 7:**
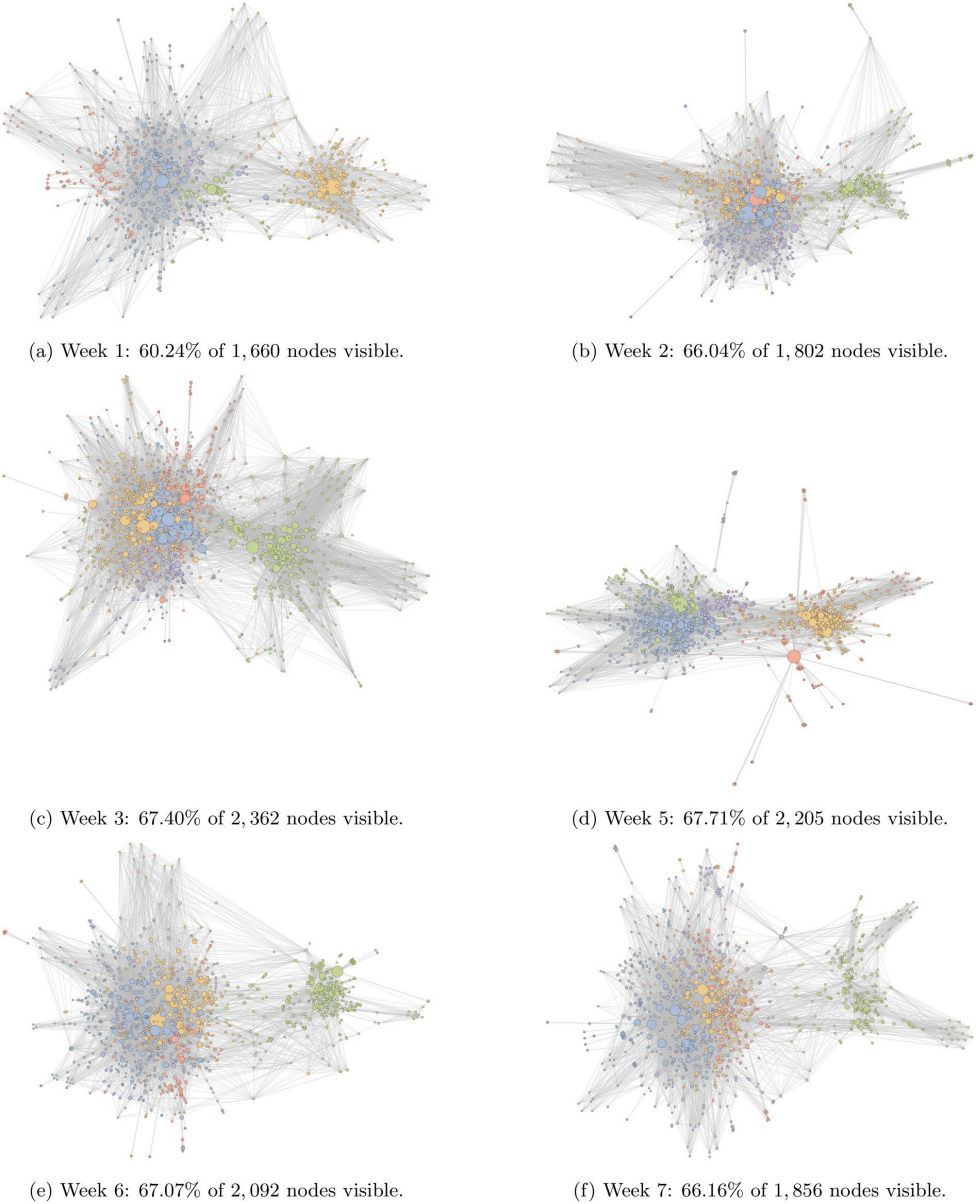
Consistent network structure over time. Network diagrams of the top five communities across the six remaining weeks. Each figure is oriented such that the left-wing cluster is on the left and the right-wing cluster is on the right. In each case node colour signifies community membership and size is proportional to the square root of total share count. Communities are labelled 1 − 5 in decreasing order of size, and colored blue, yellow, green, red and purple respectively. Node placement is determined by the Python implementation of the ForceAtlas2 algorithm [50].

To understand the consistency of the polarised information-sharing process over time, the network structure was examined along with similarity/persistence of the sets of users, source domains and shared URLs (articles) across all weeks in the study period. Similarity scores are reported in Table 3. Fig 7 presents the network diagrams of the remaining six weeks in our study period (Weeks 1 − 3 & 5 − 7).

**Table 3:** Similarity scores for users, URLs and domains between each of the seven weeks calculated using Eq (3). Similarity is directional. The similarity given in cell (4, 1) is the proportion of the users/URLs/domains in Week 4 also in seen Week 1. Cool shades indicate values smaller than the mean while warm shades indicate values greater than the mean.

Week	1	2	3	4	5	6	7	
1		0.29	0.33	0.39	0.25	0.25	0.22	
2	0.24		0.30	0.35	0.22	0.24	0.21	min.(*S*) = 0.084
3	0.19	0.21		0.38	0.20	0.19	0.18	mean.(*S*) = 0.236
4	0.08	0.09	0.15		0.11	0.10	0.09	max.(*S*) = 0.389
5	0.18	0.20	0.25	0.36		0.24	0.21	stdev.(*S*) = 0.078
6	0.19	0.22	0.26	0.35	0.26		0.25	
7	0.20	0.23	0.28	0.36	0.27	0.30		
**(a)** Asymmetric overlap of users.								
Week	1	2	3	4	5	6	7	
1		0.13	0.08	0.11	0.06	0.05	0.04	
2	0.10		0.14	0.10	0.05	0.05	0.04	min.(*S*) = 0.022
3	0.05	0.10		0.16	0.05	0.04	0.03	mean.(*S*) = 0.078
4	0.02	0.03	0.06		0.06	0.03	0.02	max.(*S*) = 0.193
5	0.04	0.05	0.06	0.19		0.16	0.07	stdev.(*S*) = 0.047
6	0.04	0.05	0.06	0.10	0.17		0.16	
7	0.04	0.04	0.05	0.09	0.09	0.19		
**(b)** Asymmetric overlap of URLs.								
Week	1	2	3	4	5	6	7	
1		0.55	0.59	0.72	0.58	0.54	0.51	
2	0.44		0.58	0.75	0.48	0.55	0.48	min.(*S*) = 0.155
3	0.34	0.41		0.76	0.42	0.42	0.39	mean.(*S*) = 0.496
4	0.15	0.20	0.29		0.23	0.22	0.18	max.(*S*) = 0.767
5	0.41	0.43	0.53	0.76		0.52	0.46	stdev.(*S*) = 0.158
6	0.41	0.52	0.56	0.76	0.56		0.53	
7	0.46	0.54	0.62	0.77	0.59	0.63		
**(c)** Asymmetric overlap of domains.								

In each week, a similar division into two clusters is observed in the information-sharing network, although the specific composition of the users and shared articles that form the network changes substantially over time. The number of communities in the left-hand and right-hand clusters varies over the weeks and the relative sizes of the different communities also change. However, each week reveals the same broad pattern of a larger left-wing/environmentalist cluster split into several smaller sub-communities, with a smaller right-wing/sceptic cluster, showing that this pattern is not an artefact of the increased activity during Week 4. Taken together with Fig 3, these results demonstrate that the pattern of network division persists over time, spanning multiple weeks of ‘normal’ activity and one exceptional week of high media activity.

Considering the inter-week similarity between user populations and the source domains and URLs they shared, Table 3 shows the inter-week similarity of unique users, URLs and website domain shares, along with the minimum, mean, maximum and standard deviation of pairwise similarity measures. The shared articles in Table 3b show the lowest similarity between weeks, with a slightly higher similarity between adjacent weeks. Intuitively, the source domains from which content was shared show higher similarity between weeks in Table 3c. User populations show limited persistence between weeks, summarised by Table 3a. The overall pattern is that, from week to week, a limited proportion of the user population and set of source domains persist, with much lower persistence in the sets of articles that are shared. This is an interesting finding with respect to the high consistency in the two-cluster network structure that is reliably observed every week. The lack of persistence in shared URLs may be explained by the general volatility of news media, where articles typically have a short lifetime (e.g. 2-3 days visibility in online sharing [13]). Moderate persistence of users and sources between weeks perhaps suggests an active core group who are present each week, with a wider group who appear less frequently.

A marked difference can be observed in the typical similarity scores for Week 4 and for other weeks. Lower similarity scores were observed for users, URLs and domains from Week 4 appearing in other weeks, while conversely, higher similarity was observed for users, URLs and domains from the other six weeks appearing in Week 4. The number of new users, URLs and domains in Week 4 also shows a stark contrast with other weeks: 70.1% of users, 82.0% of URLs and 47.9% of domains are unique to Week 4 and not present in any other week. This is strong evidence that the events of Week 4 appear to have spurred an influx of both social media participants and digital media sources (users: mean 49.3%, min. 45.6%, max. 51.2%, *σ* = 2.10%; URLs: mean 70.3%, min. 66.6%, max. 73.2%, *σ* = 6.54%; domains: mean 29.8%, min. 27.3%, max. 31.5%, *σ* = 4.21%). While web domains exhibit the most stability between weeks, URLs exhibit the least stability and users fall between these two extremes.

**Fig 8:**
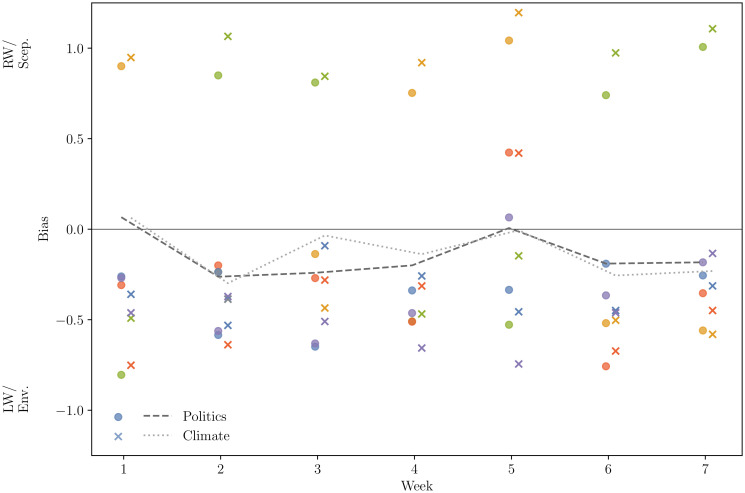
Levels of political and climate change bias over the course of the seven week study period. These are measured as the mean coded bias of domains weighted by total shares (see Ideological coding of source domains). Bias in each of the five largest communities are represented by the scatter points in each week, and the bias across the whole network is given by the lines. The colour of the community points is consistent with other figures. In most weeks, the average network bias is left of centre and more environmentalist than sceptic. This trend continues to the individual communities, with the majority being left-wing and environmentalist.

To confirm that the persistent two-cluster network structures seen in Fig 7 are polarised along the same left-right political and environmentalist-sceptic ideological axes as that seen for Week 4 (see Fig 5), the domain bias codings were applied to the networks created for Weeks 1-3 and 5-7 (S6 and S7 Figs in S1 File). Furthermore, average biases were calculated for all communities in each week in Fig 8. Trends in polarisation over time are shown as network averages for political and climate change biases, alongside the community averages, in Fig 8. In most weeks, the whole-network average shows mild left-wing and environmentalist bias. Week 5 shows (for the only time across the seven-week period) a large neutral community. Supporting the visual evidence seen in S6 and S7 Figs in S1 File, and the community-level bias scores in Fig 8, these findings show that the polarised network structure observed in Week 4 is persistent and is not an artefact of the increase in activity in the climate change conversation despite the turnover in users, source domains and shared articles.

## Discussion

This paper presents an analysis of digital media sharing behaviour around the contested issue of climate change. Our analysis looks at the network structure formed by users sharing web articles related to climate change, combining network analysis with computational and human text analyses to identify and characterise communities of users and the articles/sources they share. The aim is to understand how people engage with, and share online media content about climate change on social media (specifically Twitter).

We have found that amongst the communities of shared URLs, right-wing and climate sceptic views are strongly correlated, as are left-wing and environmentalist views. This correlation has been observed in individuals before using survey-based methods (e.g. [3, 51, 52]). Our study shows that the association extends to media outlets, specifically to the content produced by a large set of online news providers (Fig 4), and is a major feature of how online content is shared. Recent work by Hornsey et al. [52] suggests that the scepticism-conservatism link may be strongest in the US. This finding is supported here, where we observe two large communities based mainly on US sources; one more left-wing and environmentalist, the other more right-wing and sceptic. Although we did not examine where the users in our dataset were located geographically, the dominance of US sources in two of the communities, and of UK sources in one of the communities, suggests that these communities are largely formed around users from those countries. Our overall focus on English-language tweets and media content, plus large numbers of Twitter users in these countries (nearly 15% of online US adults used Twitter in 2017 [12]), are consistent with this conjecture.

In this study we find two levels of community structure. At the highest level, there is polarisation and segregation with a large left-wing/environmentalist grouping and a smaller right-wing/sceptic grouping clearly visible in the sharing network after the application of the force-directed layout algorithm. Within the left-wing/environmentalist group, algorithmic community detection finds (typically) four smaller groupings, characterised by further analysis as above. It is interesting to note that the right-wing/climate sceptic group is more densely connected internally than the left-wing/environmentalist group (Student’s *t*-test applied to average clustering coefficients of all left-wing and right-wing communities in Figs 3 and 7, with clustering coefficients calculated in Gephi using Latapy’s algorithm [53], *p* = 0.019). This finding echoes the observation by Colleoni et al. [54] that in the US, active Republicans exhibit greater levels of homophily than Democrats in their patterns of interaction on Twitter.

The temporal analysis presented above gives confidence that the polarised network structure is robust over time, despite turnover in the user populations, sets of shared articles and news sources. We studied similarity between the sets of users, URLs and domains across the seven weeks, as well as the proportion of users, URLs and domains appearing only in the exceptional Week 4 (in which climate change was the subject of a mainstream news event when President Trump announced the US withdrawal from the Paris Agreement). This major event substantially increased the volume of social media messages related to climate change and drew in an increased number of new users, who shared a greater number of articles from a greater number of online information sources. Thus we observed network structure and composition in both ‘normal operation’ as well as in an unusual state of high activity, showing the typical level of week-to-week volatility as well as the substantial change under the influence of the disruptive mainstream news event. At all times, including the disruptive event, the polarised network structure remains strong and clearly visible, despite substantial changes in its constituent parts (users and articles). As such, we conclude that the broad topological features visible in Figs 3 and 7 are stable over the seven-week duration of our dataset. Similarly, we find that the association of left-wing/environmentalist views, and of right-wing/sceptic views, are persistent, as shown in Fig 5a and 5b, S6 and S7 Figs in S1 File. Future study may find it fruitful to examine whether such stability exists for other online networks or other politicised discussions.

The similarity statistics in Table 3 suggest a general trend for a small proportion of the users, URLs and domains to persist across weeks while others appear only sporadically. In the URL case, the comparatively low levels of similarity are not surprising as news articles are quickly superseded by new facts and perspectives. Among the users and domains, the evidence suggests the network has a stable core with an unstable periphery. Over the seven weeks studied, a number of websites establish themselves as critical to the flow of information around climate change, either as news sources (such as *The Guardian* or *Breitbart*) or as conduits for personal opinions (such as *Twitter* and *Wordpress*). Many other sites are peripheral to the news-sharing network of climate change, appearing sporadically with a lower frequency of usage. This paper has deliberately avoided studying users as individuals, but it seems reasonable to expect a core group of committed and strongly interested people who regularly share information about climate change, with others who contribute less frequently or only when motivated by external factors. Considering this user behaviour in the context of the persistent network structure we have observed, it appears that the revealed polarisation is a feature of the system as a whole and not caused by specific events or users.

This article illuminates several new dimensions of the media debate around environmental politics and climate change. The findings complement previous studies that have shown climate-related echo chambers exist in direct user-user interactions (e.g. [18, 27]) by showing that similar structures also characterise patterns of information-sharing. The Twitter messages that form the sharing network studied here reflect a mechanism of news promotion and active attempts to inform others, which is not always the case in direct personal interactions. We also capture a vital additional element by exploring the network topology over time and demonstrating that the polarised structure persists, even when there is a disruptive mainstream news event. In addition, we have shown a strong correlation between political and climate-related ideological biases in news production and consumption, with associations between left-wing/environmental and right-wing/sceptic positions. This widens the scope of previous studies of mainstream news media (e.g. [51]) to include the increasingly important online news media.

When looked at in isolation, several domains appear to be coded with more of a left-wing, environmentalist bias than would be expected by domain knowledge experts. Typically neutral sites, such as Bloomberg, are reported as left-wing and environmentalist primarily through the lenses they use to cover the specific topic of climate change. Other domains, such as the Daily Caller, may have been poorly represented by the content sampling process as factual and scientific extracts taken without surrounding challenging context were considered as supporting the scientific consensus. This “good faith” on the part of the coders returns a particularly erroneous response for Watts Up With That, a prominent blog in the sceptical community. Many of their articles present quotations from public figures and scientific papers in addition to their own commentary that frequently challenges the framing, which are contextual clues that may be unavailable to the coders. The last explanation for possible misclassification of certain domains identified in this exercise is the reposting of content from other sources. Investigation of the articles coded from Fox News found that three of the five articles were from Associated Press sources, which may present a different editorial bias to Fox News original content. The choice of which content to repost from other sources is an important editorial decision and as such we believe this inclusion has not adversely affected our findings.

Future work in this area could further examine the ties between political and environmental opinions. This study used human coders to detect biases from article content. While this approach was successful, it carries substantial costs which make it hard to operate for large datasets. It could be argued that our use of subjective human grading is a limitation in our analysis, but it is a necessary compromise given the inherently subjective nature of political and environmental beliefs and the current lack of objective tools for analysing such complex interpretations in large quantities of data. An automated classifier for these biases would require significant work for its creation (using a blend of machine learning and natural language processing), but would support future large-scale studies, a necessary step given the ever-increasing volume of online content. Another research question concerns whether the patterns observed on Twitter extend to other online platforms. We studied Twitter due to its prominent role as a means for users to find news stories [55] and its frequent usage as a platform for lively political debates in which strangers can interact. If suitable datasets could be obtained, it would be possible to apply similar methods to other popular sites such as Facebook and Reddit; however, privacy restrictions prevent research on many social media platforms. Finally, one further question that is not addressed here is downstream exposure to shared content, that is, who sees the articles that are shared by Twitter users? It is probable that the users captured in our dataset, that select and share online articles about climate change through Twitter, act as ‘opinion leaders’ (following the long-established two-step flow model of Katz and Lazarsfeld [56]), locating new information about the topic and disseminating it to their followers. Measuring the volume of downstream views and retweet rates would answer important questions about which messages are more effective when shared on social media. One key question is the extent to which polarisation exists in this secondary consumption of climate change media, or whether network effects mitigate polarisation by capturing views from both sides.

Any gathering of data from online social networks requires careful consideration of the biases that it may introduce. The users of online platforms are a different distribution of people than the general population: they tend to be younger, more wealthy and better educated [57]. Our sampling on keywords means that we will capture users who are more engaged with the topic of climate change, and moreover are significantly invested enough to share information with their followers. As such we are considering a highly-motivated sample of a certain part of society, but this is what will be visible to many users when they visit Twitter. This sampling also explains the difference from Global Warming’s Six Americas [1] that we observe, as only the “alarmed” and “dismissive” types are likely to be invested enough to be captured. The work of Williams et al. [18] support this as their coding of Twitter users found only the extreme environmentalists and sceptics. Our keywords were chosen to capture as much of the English language components of the climate change conversation online, but the observed structures may vary in other languages. These factors should be considered in any analysis of social media data but we do not believe that they affect the strength of our findings in any way.

This work demonstrates that media communication of information around climate change faces many challenges in the age of social media. Users return to the same trusted sources for information even when presented with new contexts, and any attempts to attract new readerships need to consider this behaviour. Understanding how these trusted ties form will be key to combating the spread of misinformation that currently challenges online social networks and vital for promoting action on climate change and other societal challenges.

## Supporting information

S1 File (PDF)
