# Supplementary material for: Ideological biases in social sharing of online information about climate change
Source: PLoS One. 2021 Apr 23;16(4):e0250656. doi: 10.1371/journal.pone.0250656 (PMC8064565; doi:10.1371/journal.pone.0250656)
Supplement: S1 File — (PDF) [file pone.0250656.s001.pdf]

Supporting information to Ideological biases in social sharing of online information about climate change

Tristan J.B. Cann*, Iain S. Weaver and Hywel T.P. Williams
*tc471@exeter.ac.uk

January 22, 2021

Persistence in the giant component

Restricting the analysis of persistence to the giant component in each week reduces the mean and variance but does not alter relative trends (user mean 0.231, user $\sigma = 0.084$, URL mean 0.053, URL $\sigma = 0.049$, domain mean 0.486, domain $\sigma = 0.175$). Considering only the weeks surrounding Week 4 does not noticeably alter the mean, but does reduce the variance (user mean 0.236, user $\sigma = 0.039$, URL mean 0.076, URL $\sigma = 0.045$, domain mean 0.502, domain $\sigma = 0.074$.

References

[1] Jacomy M, Venturini T, Heymann S, Bastian M. ForceAtlas2, a Continuous Graph Layout Algorithm for Handy Network Visualization Designed for the Gephi Software. PLOS ONE. 2014;9(6):1–12.

Week	1	2	3	4	5	6	7
Users	13,017	16,553	22,496	54,347	19,033	17,574	14,252
URLs	6,117	6,560	7,871	20,880	8,529	7,652	7,272
Bipartite edges	17,990	22,565	31,831	80,009	25,165	23,701	19,702
Users in giant component	8,358	11,647	17,222	42,113	12,068	11,762	8,703
URLs in giant component	1,660	1,802	2,362	7,496	2,205	2,092	1,856
Bipartite edges in giant component	12,155	16,303	24,812	63,755	16,912	16,677	12,910
Unipartite edges	10,001	12,073	17,539	53,652	12,635	11,685	11,101
Modularity	0.606	0.564	0.549	0.578	0.613	0.566	0.595
Number of communities	44	37	57	117	44	45	44

S1 Table: Summary statistics for the networks across each of the seven weeks. In each case, the number of edges represents distinct edges and modularity is given to three decimal places.

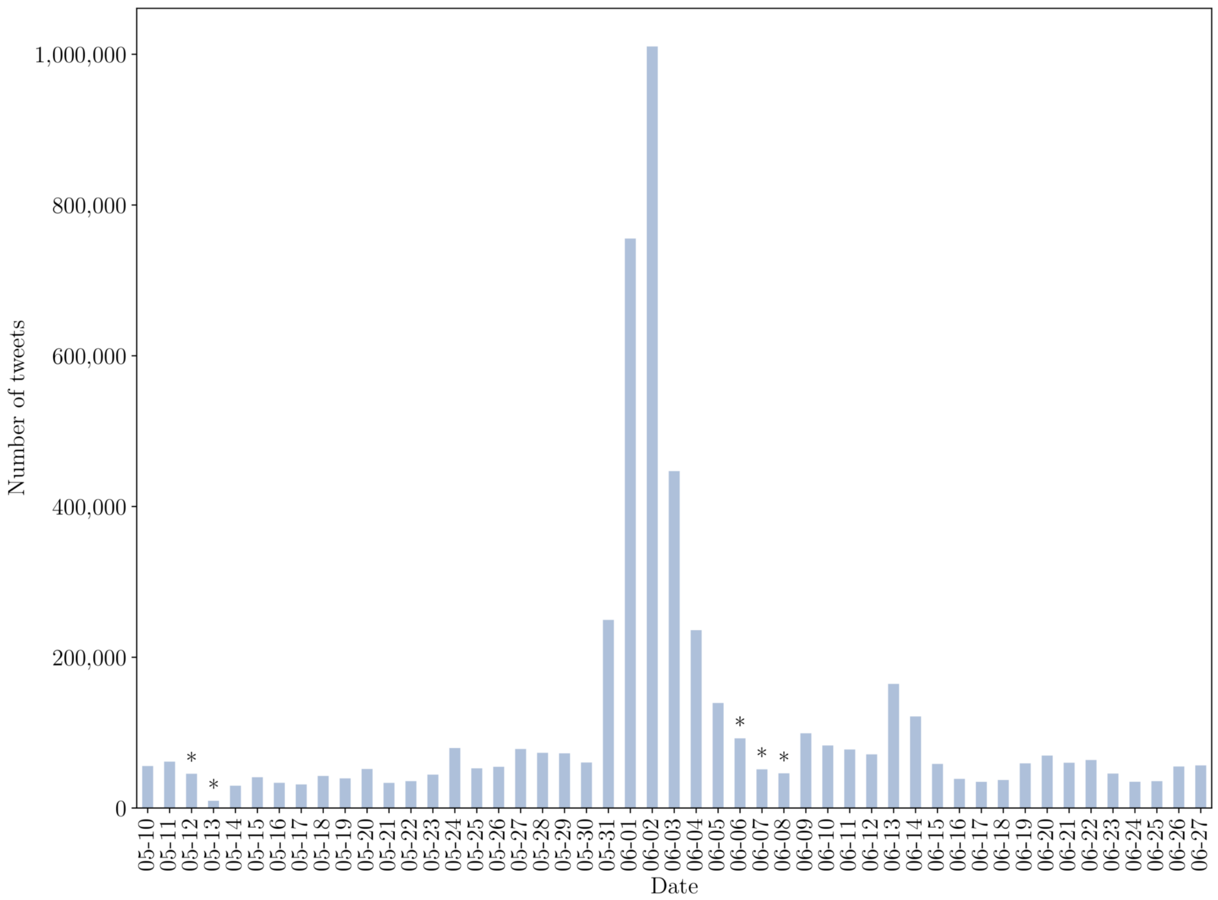

S1 Fig: Timeseries of the number of tweets per day across the seven week study period. There were a few short collection outages (11:00 2017-05-12 - 11:00 2017-05-13, 17:00 2017-06-06 - 09:00 2017-06-07 and 20:00 2017-06-07 - 10:00 2017-06-08) but since these outages represent a small proportion (around 5%) of the total collection period and mostly occurred at night, it is not expected that this has affected the validity of the experiments. Days with collection interruptions are marked with $*$. Note the increase in the number of tweets per day centered on 2017-06-02, and the evidence of weekly periodicity, particularly towards the end of June.

Week	Size of five largest communities				
1	423	264	148	123	93
2	326	271	254	212	127
3	528	354	303	209	198
4	1,732	1,263	954	762	629
5	524	391	237	201	140
6	448	350	347	139	119
7	347	264	257	200	160

S2 Table: Number of URL nodes in each of the five largest communities for each week in the study period.

Rating	-1	0	1	NaN
Political bias	L	N	R	U
Climate bias	E	N	S	U

S3 Table: Ideological coding scheme applied to each domain by the coders. This table uses the abbreviations L for Left, R for Right, E for Environmentalist, S for Sceptic, N for Neutral and U for Unclear.

theguardian.com 18,557/1,031	independent.co.uk 14,715/455	nytimes.com 12,603/535
washingtonpost.com 6,540/395	breitbart.com 4,172/205	twitter.com+ 3,949/1,364
youtube.com* 3,895/30	bbc(.co.uk or .com) 3,270/365	wordpress.com+ 2,544/1,719
dailycaller.com 2,535/228	insideclimatenews.org 2,345/157	wired.com 2,042/70
cbc.ca 1,831/232	foxnews.com 1,801/159	forbes.com 1,772/174
nationalgeographic.com 1,766/186	thehill.com 1,748/140	thegatewaypundit.com 1,699/44
bloomberg.com 1,633/193	thinkprogress.org 1,595/150	ecowatch.com 1,560/103
theconversation.com 1,526/115	politico.com 1,516/68	qz.com 1,373/87
theblaze.com 1,351/63	mashable.com 1,306/94	newslocker.com* 1,300/1,297
theatlantic.com 1,265/87	time.com 1,158/97	motherjones.com 1,135/110
ijr.com 1,132/28	scientificamerican.com 1,131/192	fastcompany.com 1,091/37
dailymail.co.uk 1,063/204	change.org 1,056/142	facebook.com+ 1,043/519
nasa.gov 1,032/99	zerohedge.com 1,030/52	futurism.com 1,026/83
naturalnews.com 984/67	infowars.com 916/46	telegraph.co.uk 913/150
cnn.com 913/197	newsweek.com 904/87	truthfeed.com 902/31
usatoday.com 892/96	foreignpolicy.com 844/36	iflscience.com 836/56
thetruthdivision.com 814/3	territoryairservices.com* 811/147	latimes.com 805/140
abc.net.au 791/145	rt.com 775/44	medium.com 767/223
buzzfeed.com 741/51	dailykos.com 710/95	prageru.com* 695/10
rightrelevance.com* 694/1	dailywire.com 661/50	reddit.com+ 657/393
reuters.com 642/208	fw.to* 600/162	trendolizer.com 598/595
newyorker.com 580/44	wattsupwiththat.com 568/138	weather.com 561/105
wsj.com 538/92	yournewswire.com 517/33	grist.org 514/111
vice.com 509/105	economist.com 508/34	investors.com 501/29
google.com* 494/56	paper.li* 489/144	weforum.org* 477/74

S4 Table: The 75 most common domains by share count across the seven weeks of the study period. These domains are ordered by total share count. Numbers indicate total number of shares/number of unique articles. * denotes the domains excluded from analysis due to incompatibility with the Diffbot API. + denotes the domains excluded as social media sites. Note that the number of unique URLs for youtube.com and google.com are artificially low due to the disambiguation step removing video identifiers.

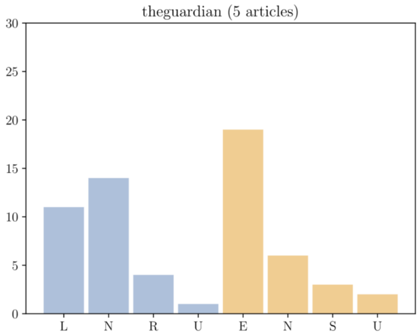

The Guardian

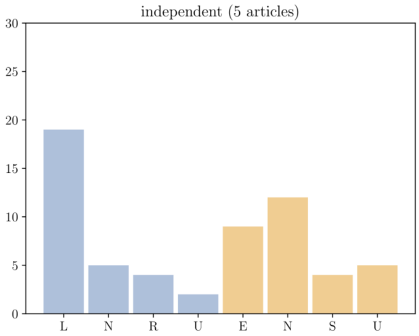

Independent

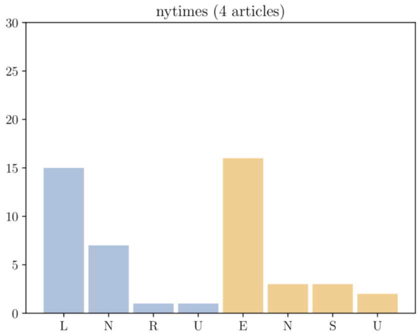

New York Times

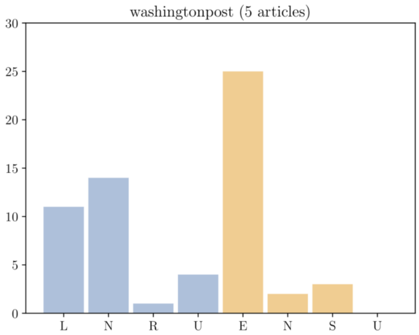

Washington Post

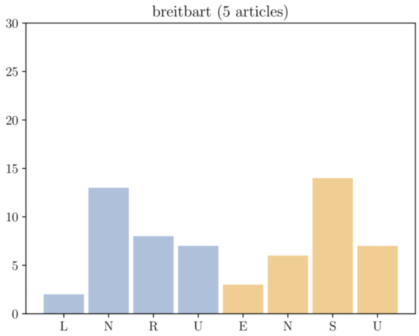

Breitbart

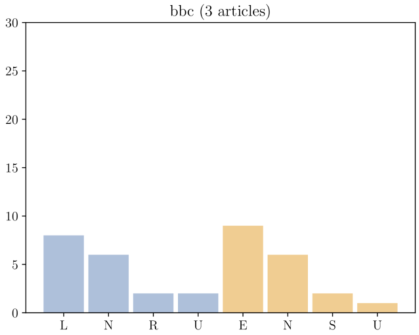

BBC

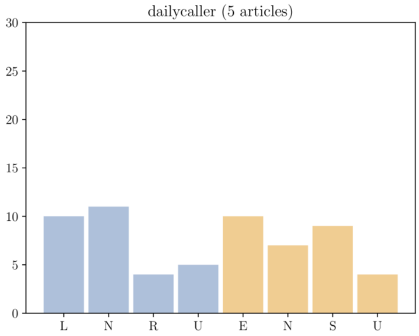

Daily Caller

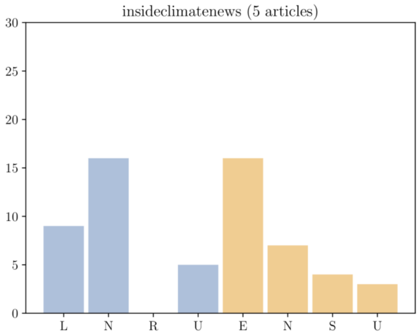

Inside Climate News

S2 Fig: Bias grades assigned by the coders to each domain.

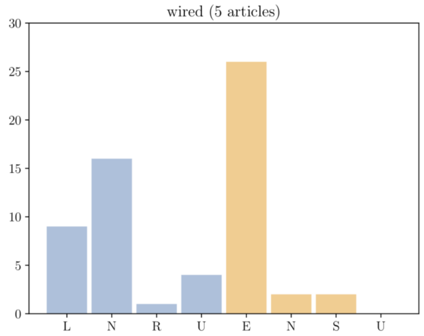

Wired

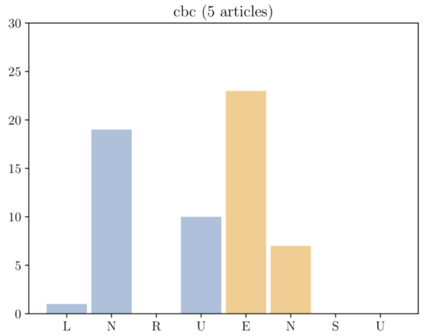

CBC

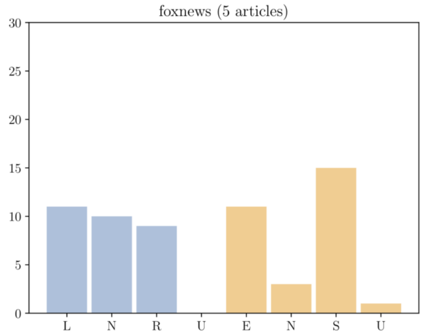

Fox News

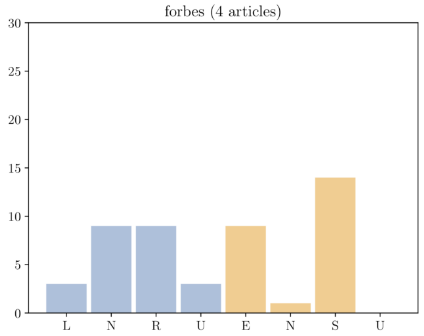

Forbes

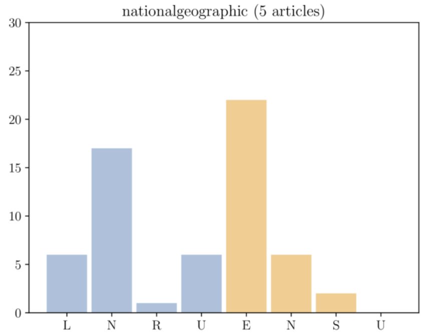

National Geographic

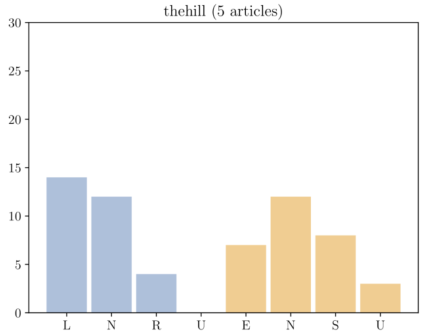

The Hill

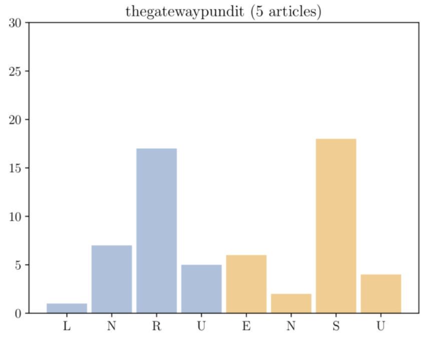

The Gateway Pundit

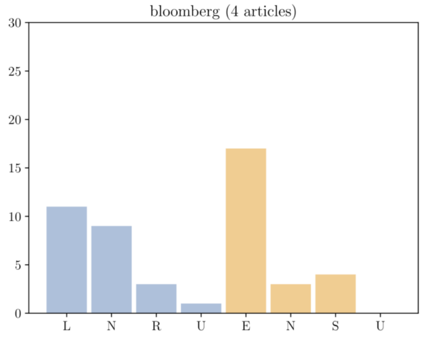

Bloomberg

S2 Fig (Cont.): Bias grades assigned by the coders to each domain.

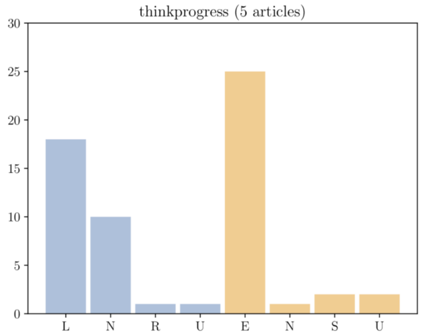

Think Progress

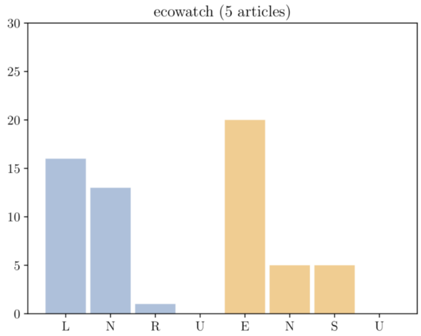

Ecowatch

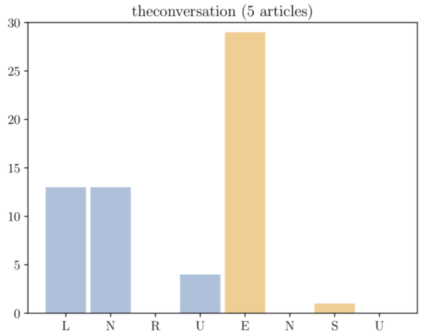

The Conversation

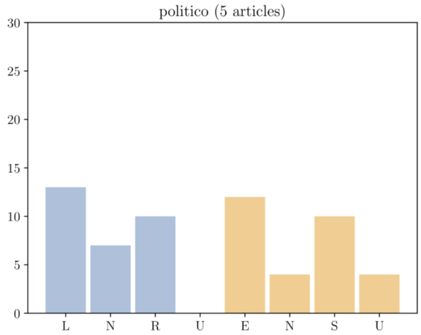

Politico

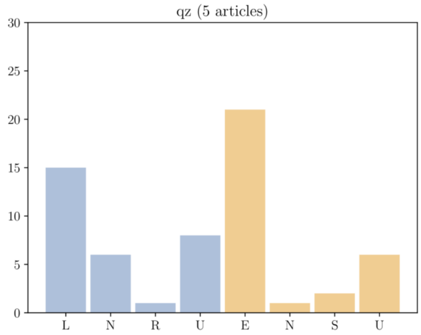

Qz

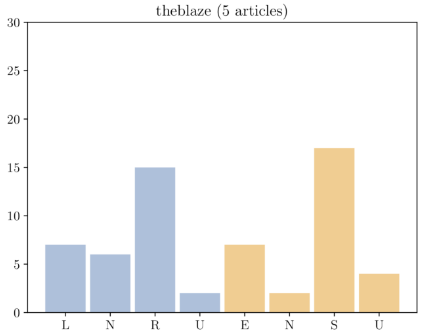

The Blaze

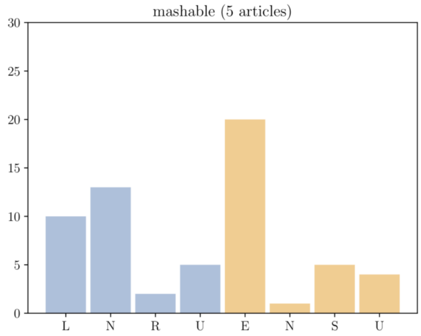

Mashable

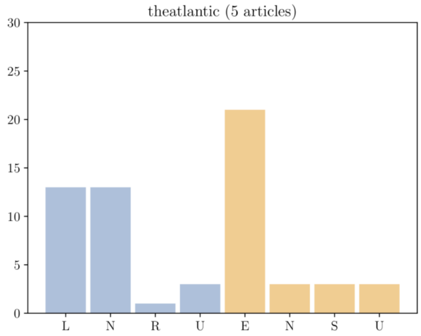

The Atlantic

S2 Fig (Cont.): Bias grades assigned by the coders to each domain.

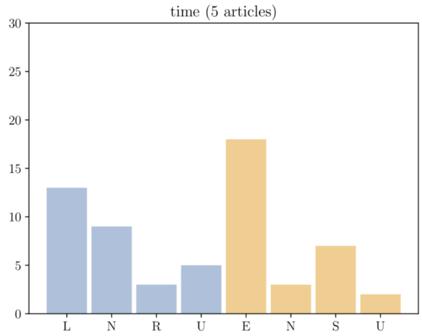

Time

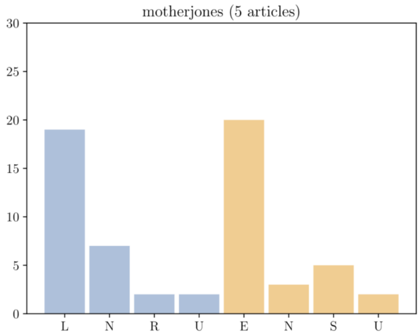

Mother Jones

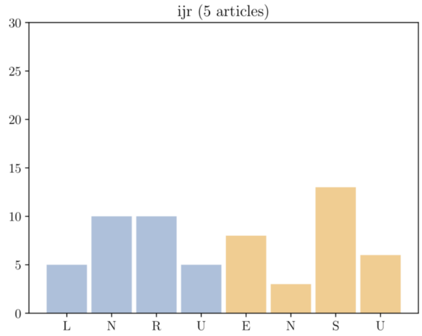

IJR

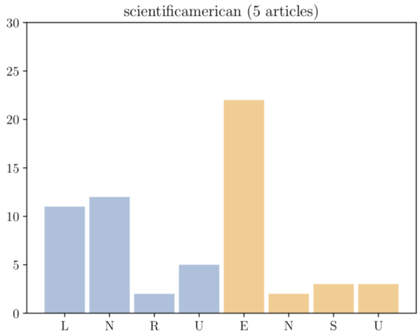

Scientific American

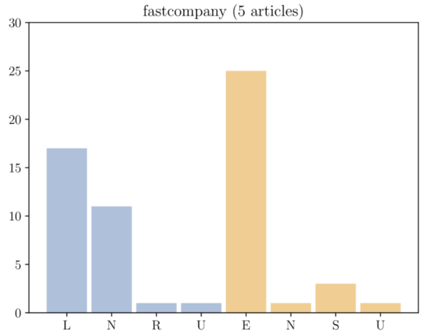

Fast Company

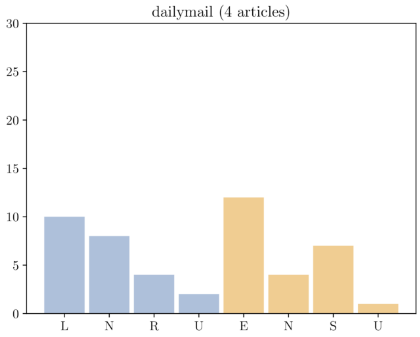

Daily Mail

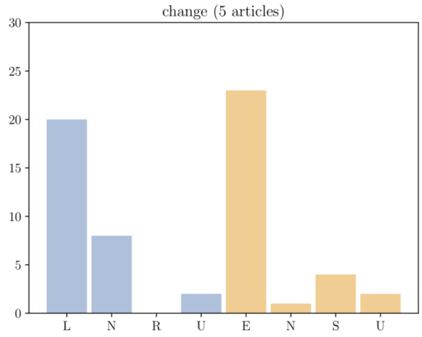

Change

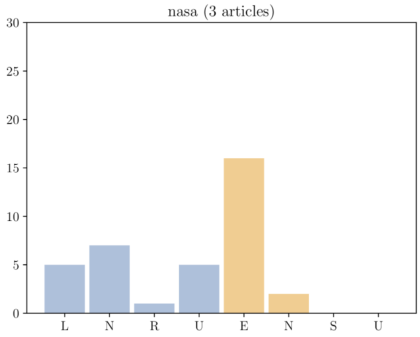

NASA

S2 Fig (Cont.): Bias grades assigned by the coders to each domain.

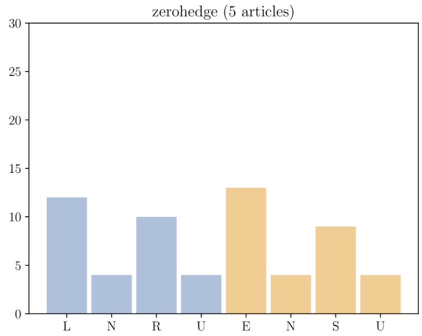

Zero Hedge

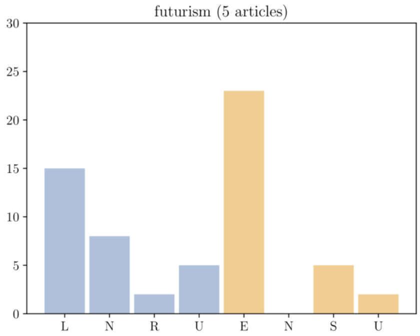

Futurism

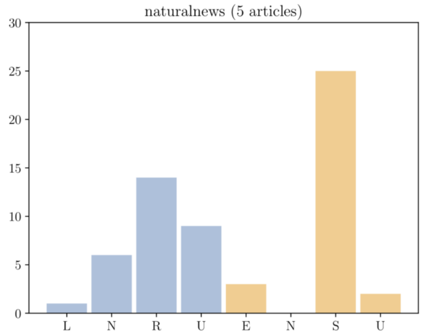

Natural News

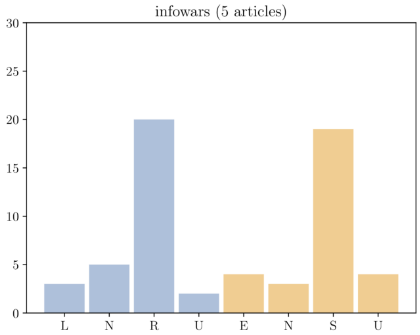

Info Wars

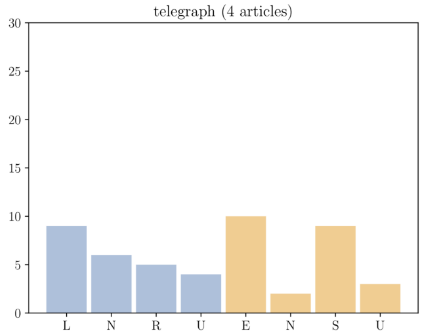

Telegraph

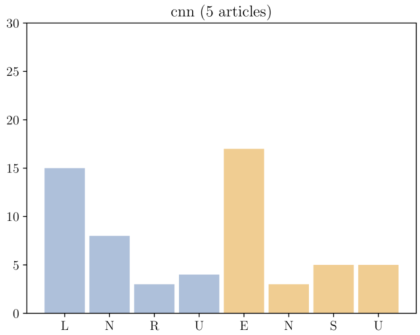

CNN

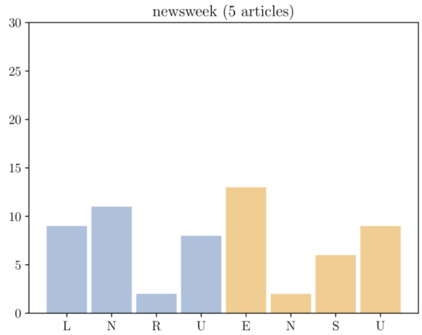

Newsweek

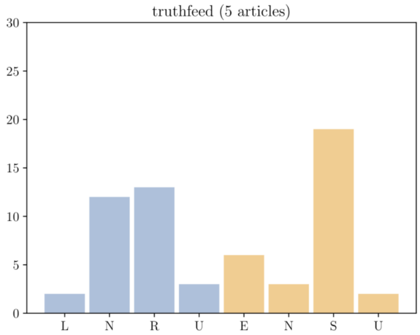

Truthfeed

S2 Fig (Cont.): Bias grades assigned by the coders to each domain.

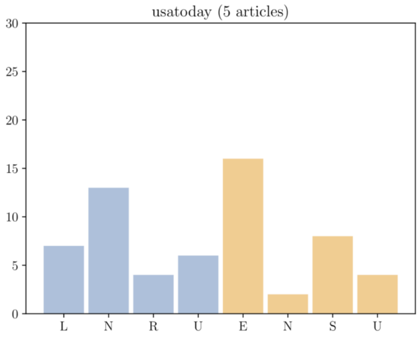

USA Today

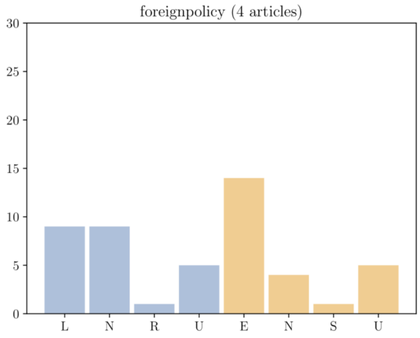

Foreign Policy

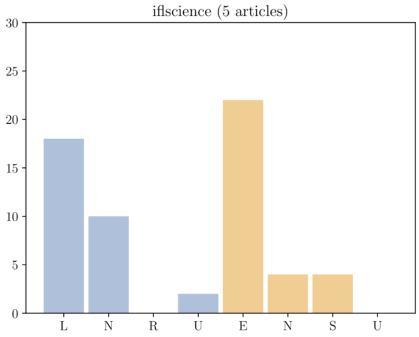

IFL Science

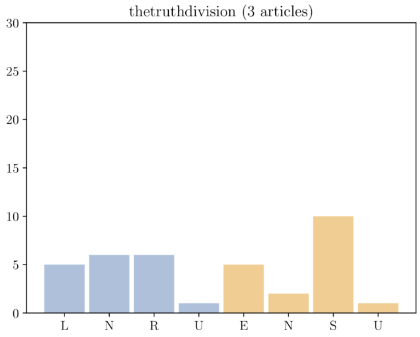

The Truth Division

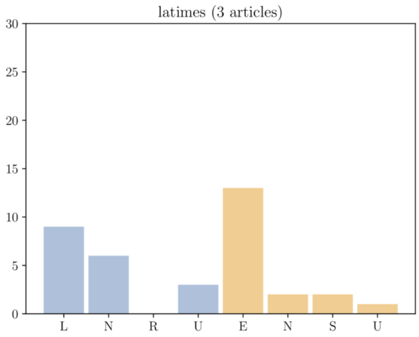

LA Times

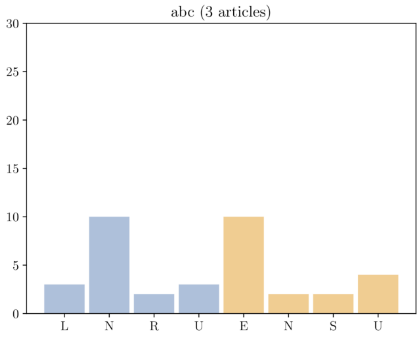

ABC

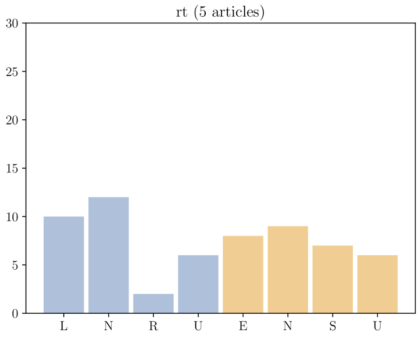

RT

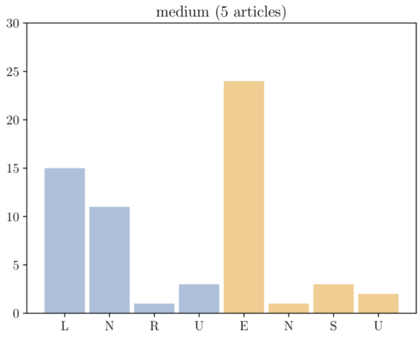

Medium

S2 Fig (Cont.): Bias grades assigned by the coders to each domain.

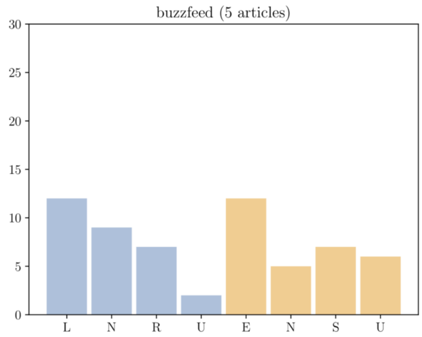

Buzzfeed

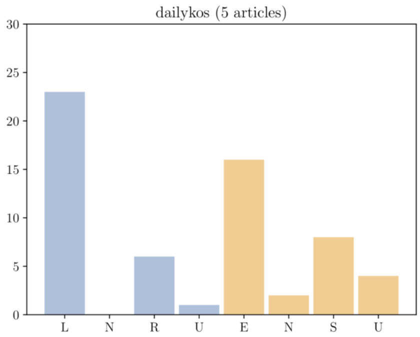

Daily Kos

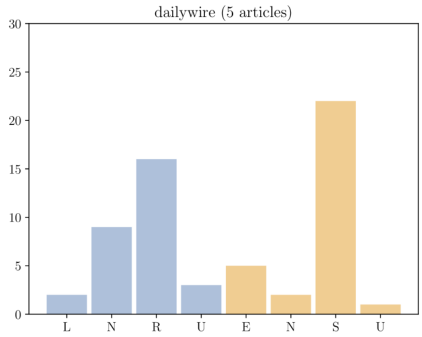

Daily Wire

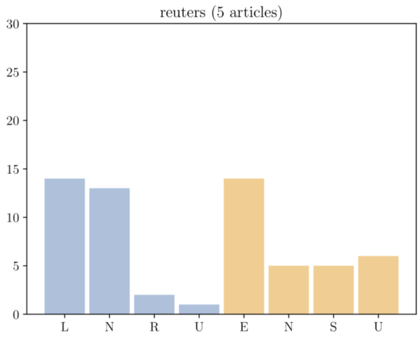

Reuters

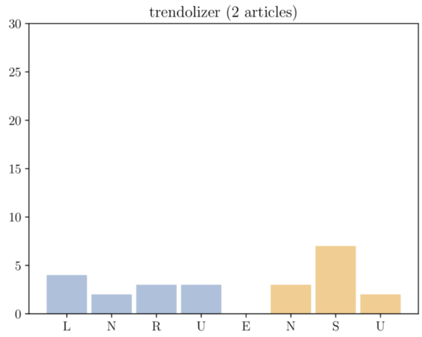

Trendolizer

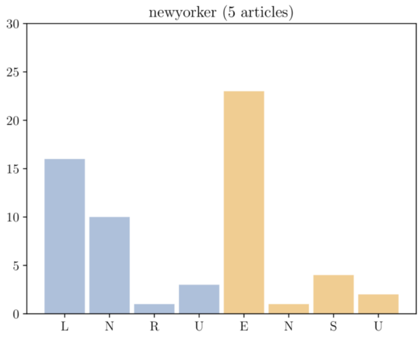

New Yorker

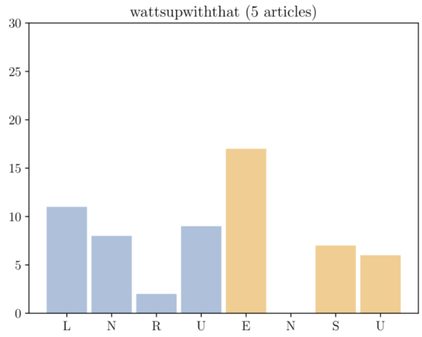

Watts Up With That

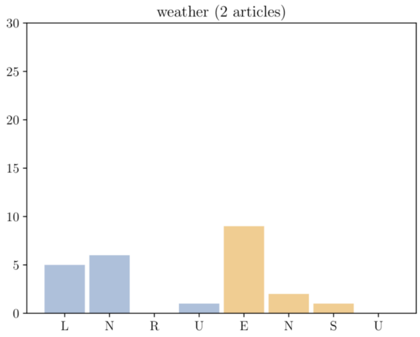

Weather

S2 Fig (Cont.): Bias grades assigned by the coders to each domain.

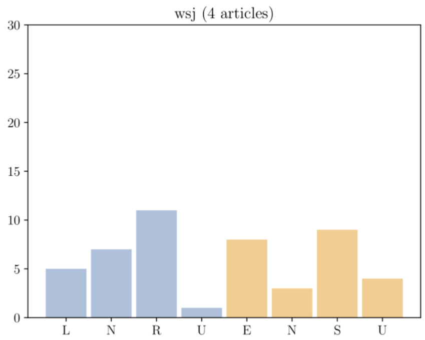

WSJ

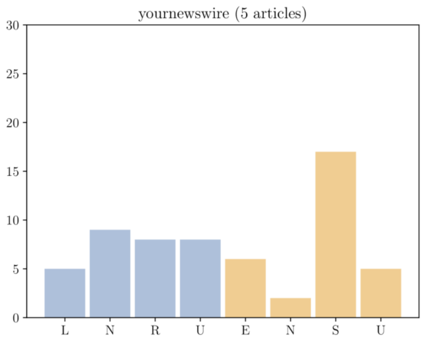

Your News Wire

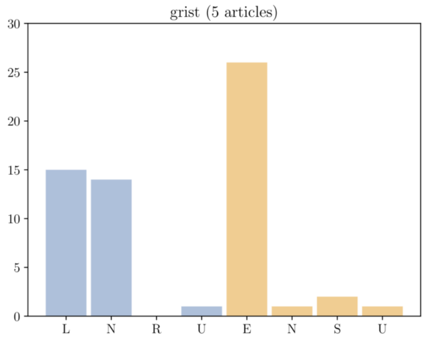

Grist

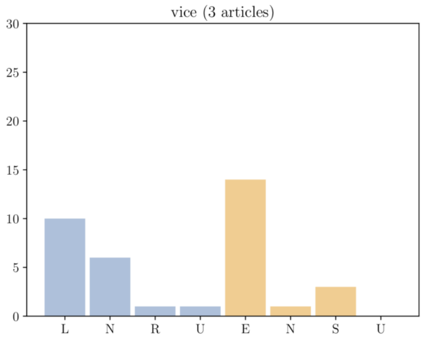

Vice

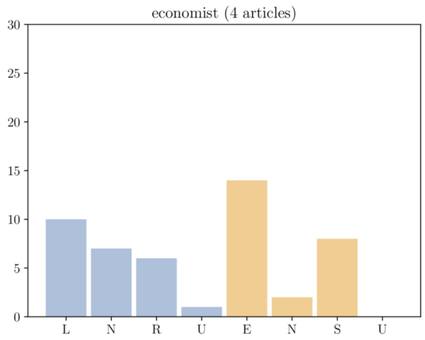

Economist

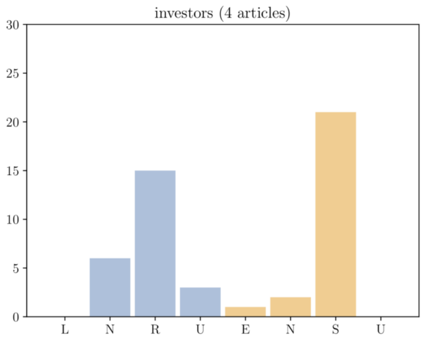

Investors

S2 Fig (Cont.): Bias grades assigned by the coders to each domain.

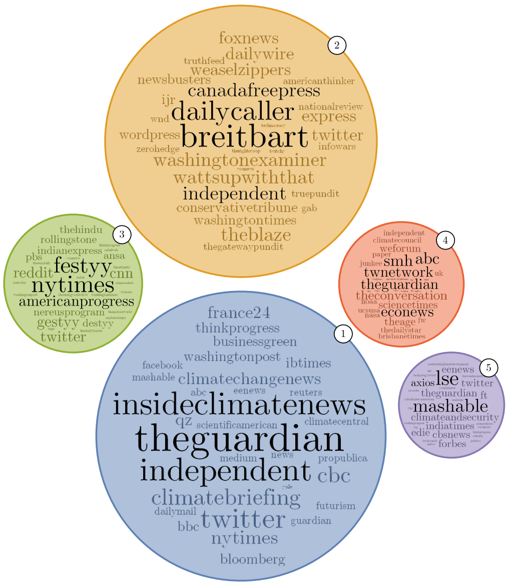

(a) Week 1

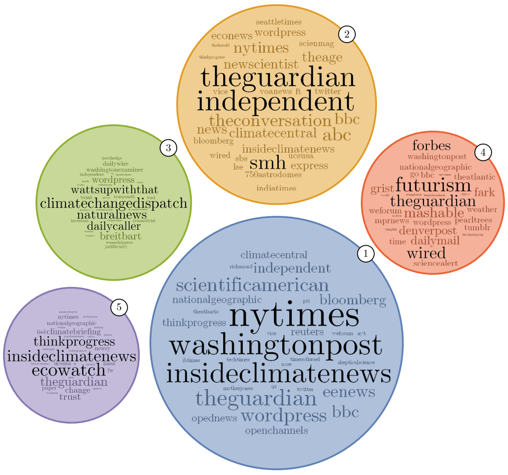

(b) Week 2

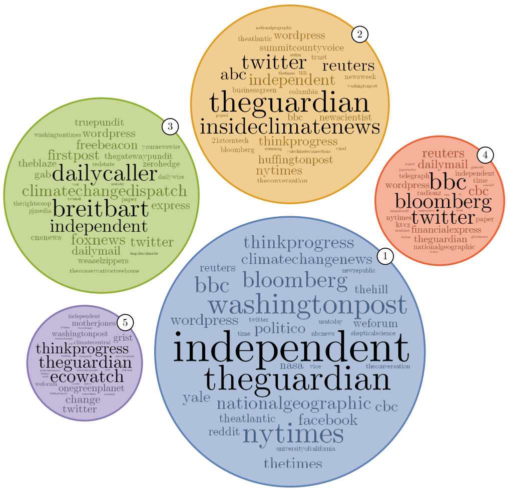

(c) Week 3

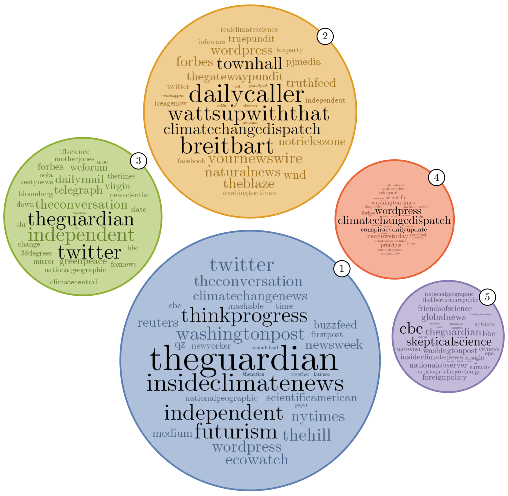

(d) Week 5

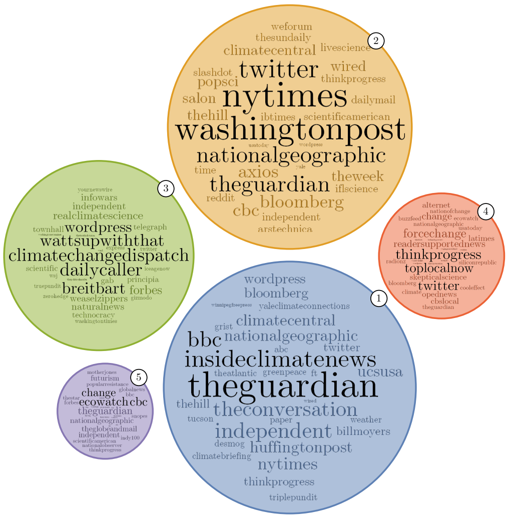

(e) Week 6

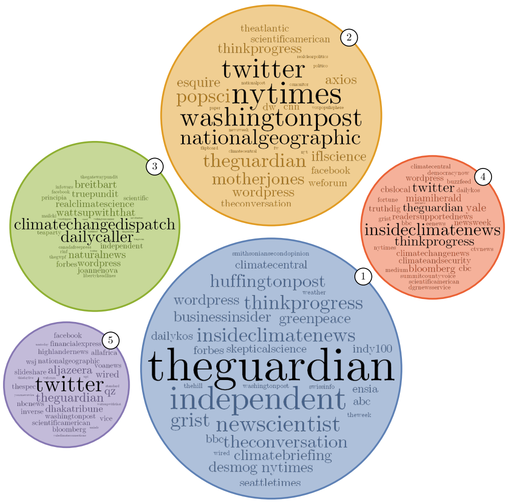

(f) Week 7

S3 Fig: TF-IDF weighted domain wordclouds for the five largest communities by share count over the remaining six weeks. Circle size is determined by the total number of shares for all URLs in the community. Terms coloured black are the highest weighted terms required to reach 15% of the total weight.

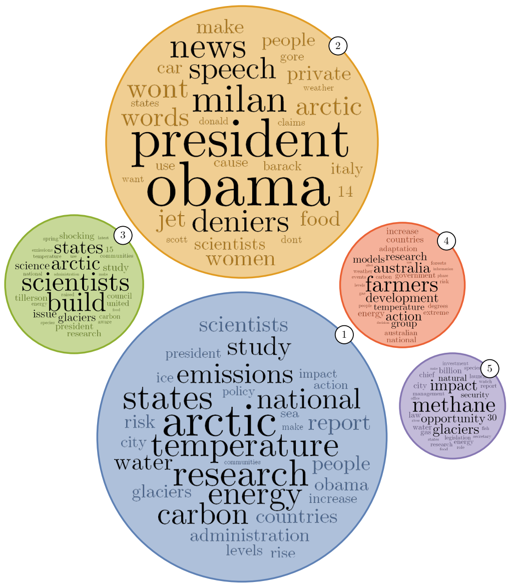

(a) Week 1

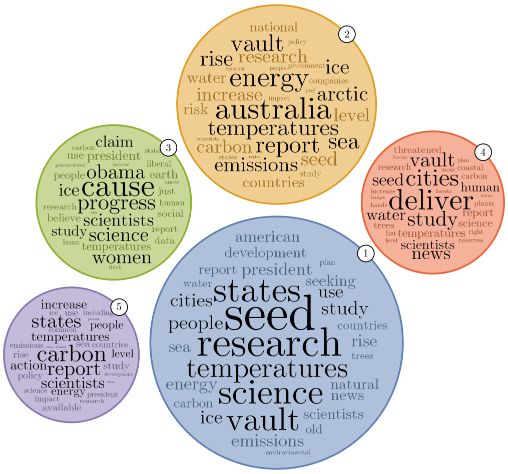

(b) Week 2

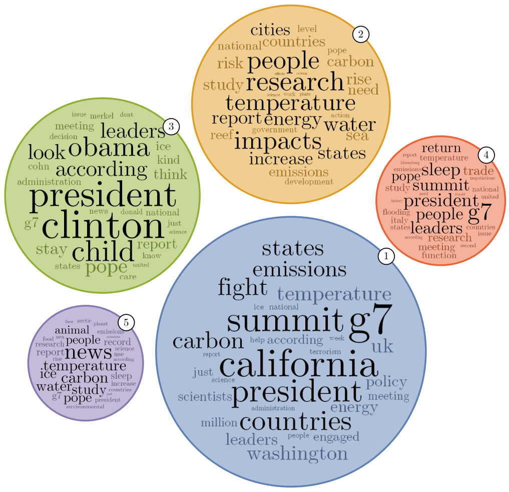

(c) Week 3

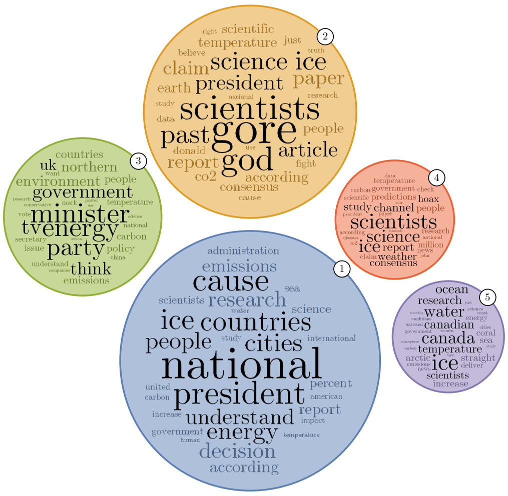

(d) Week 5

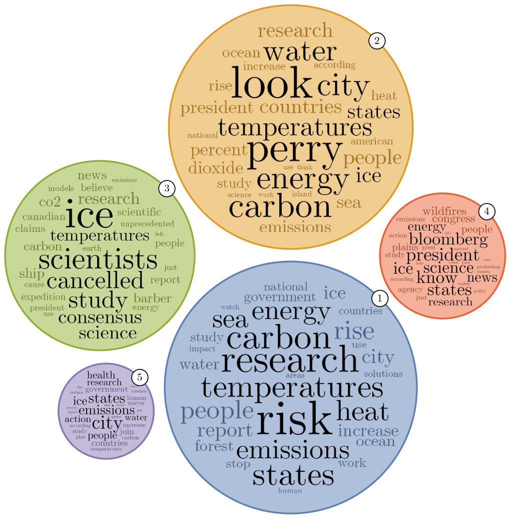

(e) Week 6

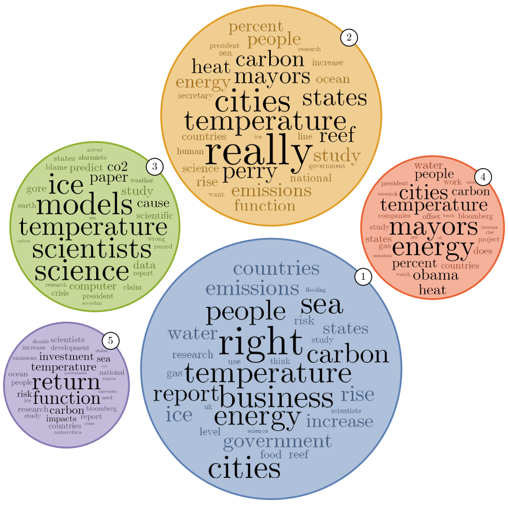

(f) Week 7

S4 Fig: TF-IDF weighted content wordclouds for the five largest communities by share count over the remaining six weeks. Circle size is determined by the total number of shares for all URLs in the community. Terms coloured black are the highest weighted terms required to reach 15% of the total weight.

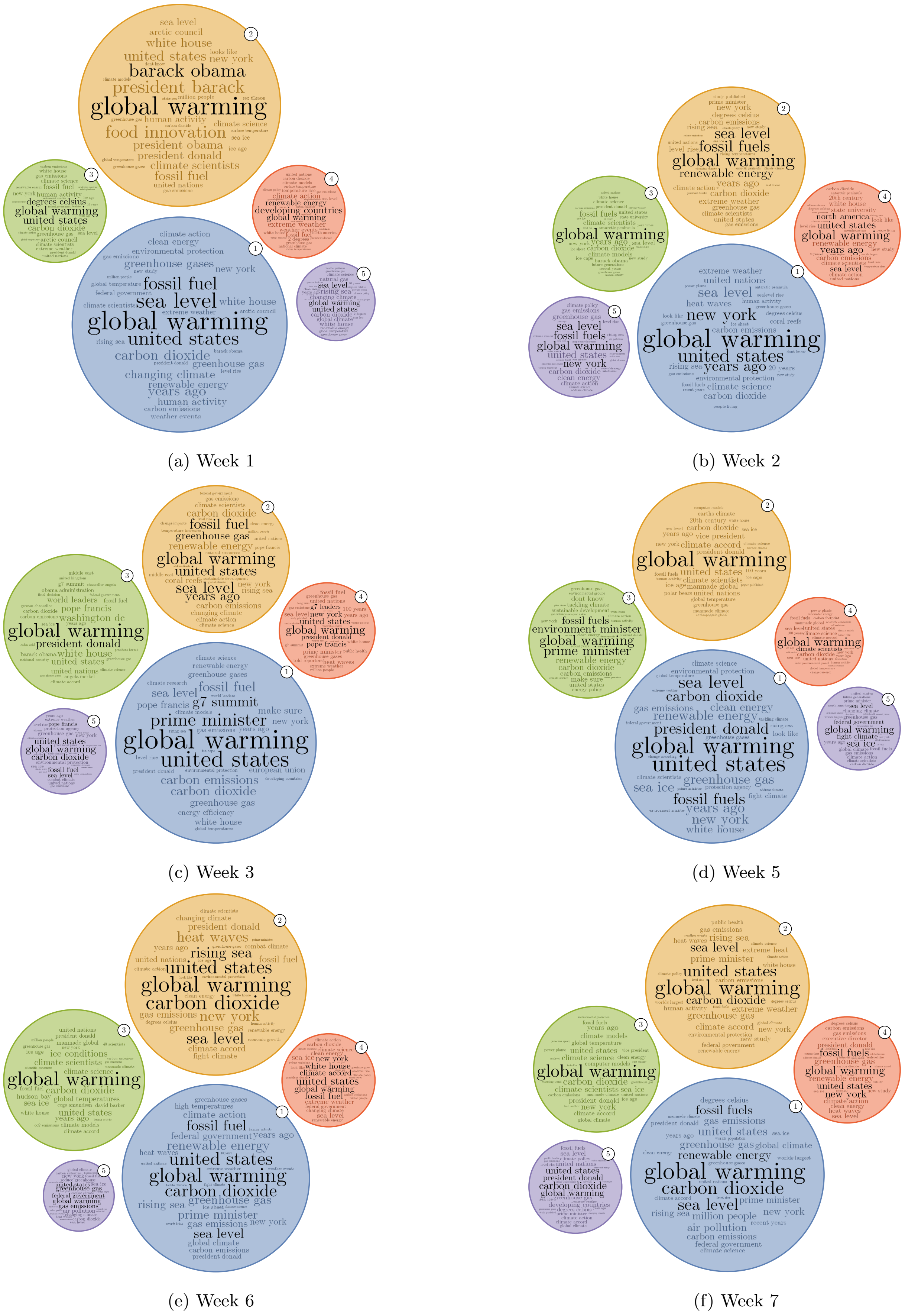

(a) Week 1 (b) Week 2

(c) Week 3 (d) Week 5

(e) Week 6 (f) Week 7

S5 Fig: TF-IDF weighted content bigram wordclouds for the five largest communities by share count over the remaining six weeks. Circle size is determined by the total number of shares for all URLs in the community. Terms coloured black are the highest weighted terms required to reach 15% of the total weight.

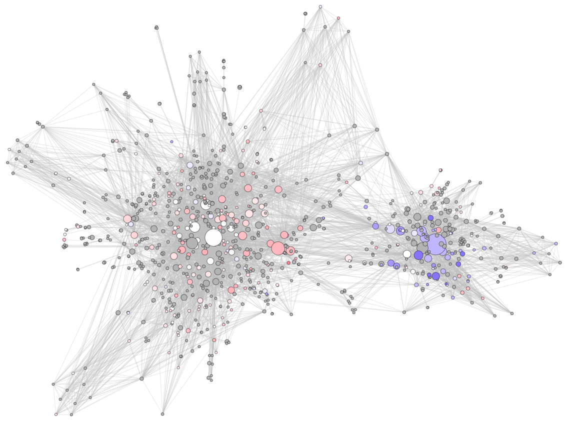

(a) Week 1: 60.24% of 1,660 nodes visible.

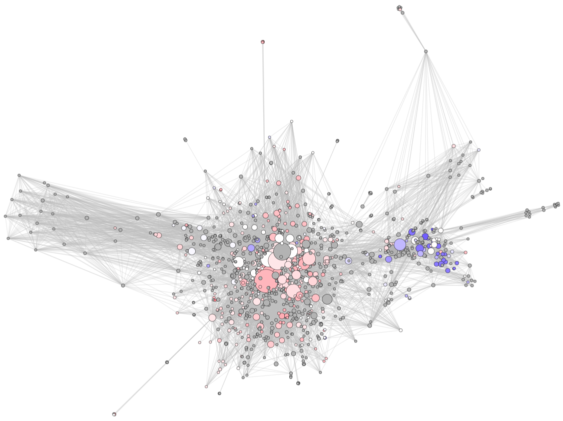

(b) Week 2: 66.04% of 1,802 nodes visible.

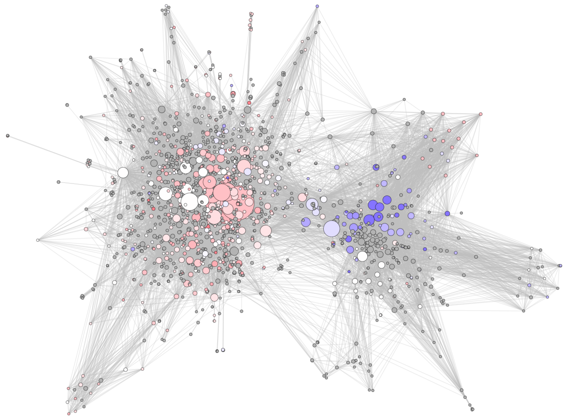

(c) Week 3: 67.40% of 2,362 nodes visible.

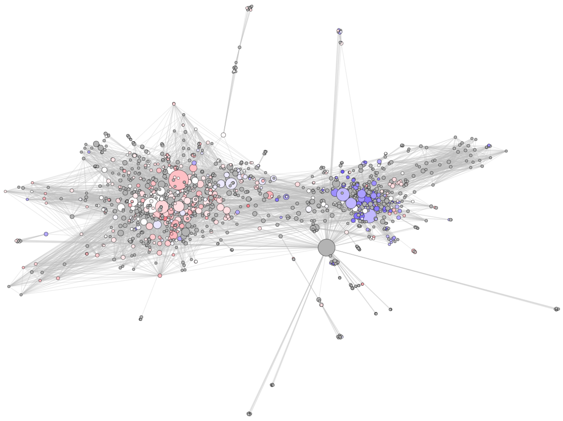

(d) Week 5: 67.71% of 2,205 nodes visible.

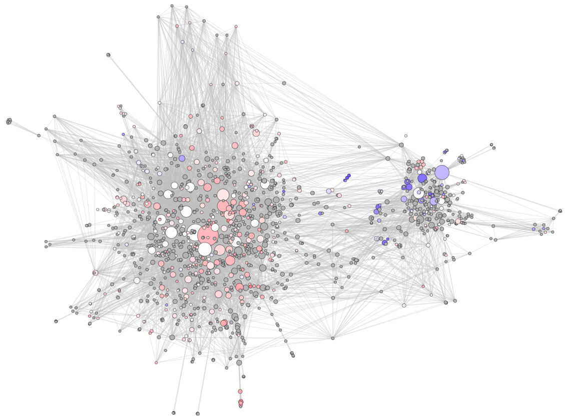

(e) Week 6: 67.07% of 2,092 nodes visible.

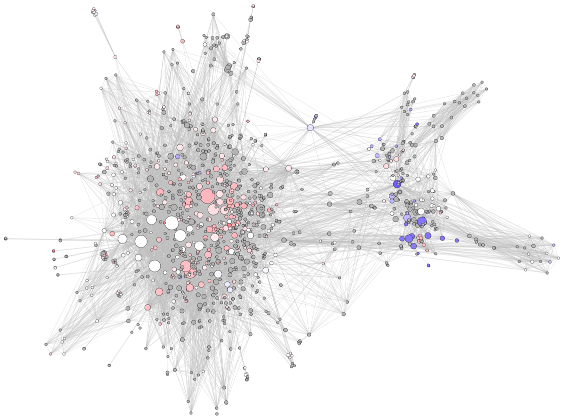

(f) Week 7: 66.16% of 1,856 nodes visible.

S6 Fig: Network diagrams of the top five communities across the six remaining weeks. Each figure is oriented such that the left-wing cluster is on the left and the right-wing cluster is on the right. In each case node colour signifies the political bias of domains as determined by the team of coders and size is proportional to the square root of total shares. Red nodes are from left-wing sources and blue nodes are from right-wing sources. Any node coded as neutral is white and gray indicates uncoded domains. Node placement is determined by the Python implementation of the ForceAtlas 2 algorithm [1]. The pattern of bias split between the clusters is consistent across the study period.

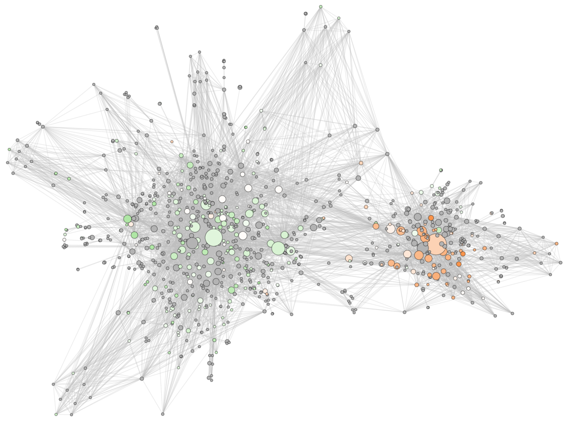

(a) Week 1: 60.24% of 1,660 nodes visible.

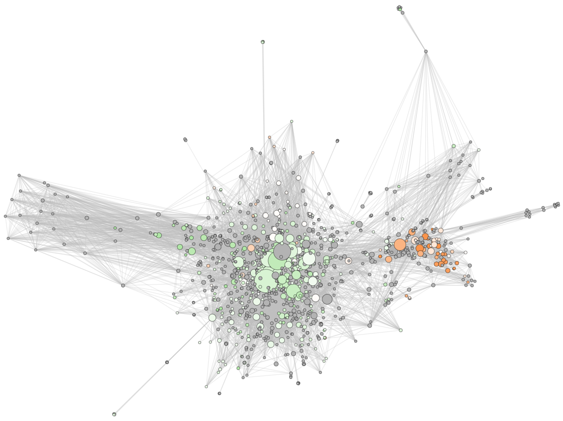

(b) Week 2: 66.04% of 1,802 nodes visible.

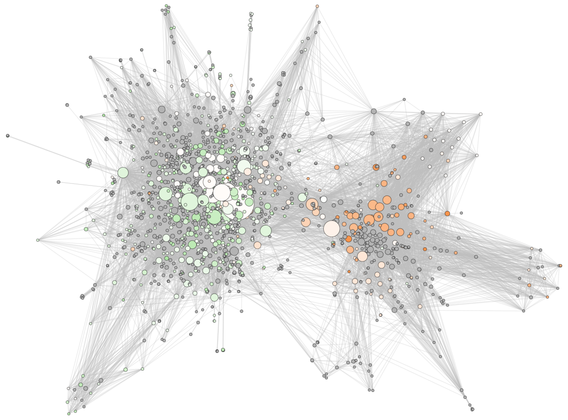

(c) Week 3: 67.40% of 2,362 nodes visible.

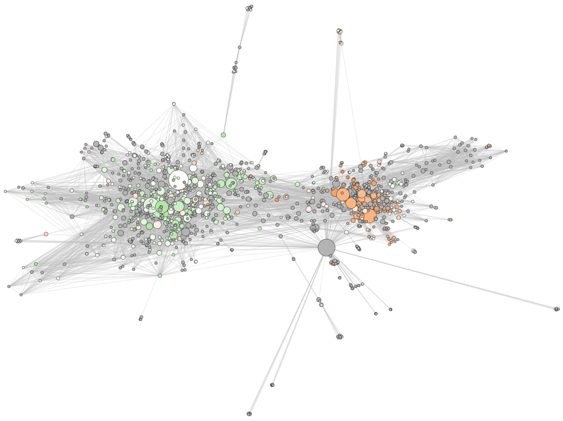

(d) Week 5: 67.71% of 2,205 nodes visible.

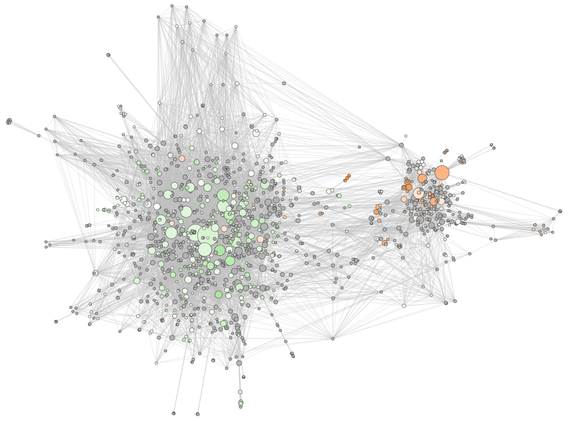

(e) Week 6: 67.07% of 2,092 nodes visible.

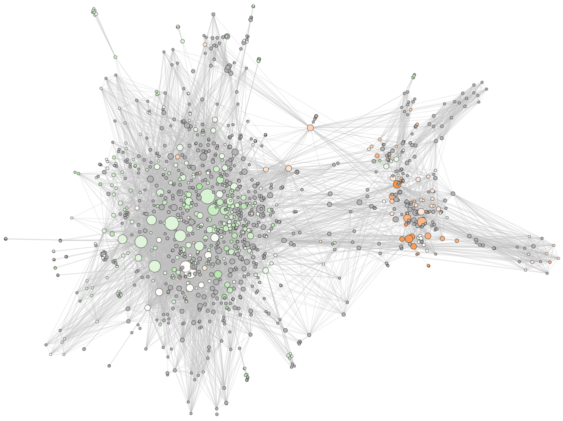

(f) Week 7: 66.16% of 1,856 nodes visible.

S7 Fig: Network diagrams of the top five communities across the six remaining weeks. Each figure is oriented such that the left-wing cluster is on the left and the right-wing cluster is on the right. In each case node colour signifies domain bias around climate change as determined by the team of coders and size is proportional to the square root of total shares. Green nodes are from environmentalist sources, orange nodes are from sceptic sources and any nodes from domains coded as neutral are white and gray indicates uncoded domains. Node placement is determined by the Python implementation of the ForceAtlas 2 algorithm [1]. Each week reveals the same pattern of polarisation in the network and demonstrates it is stable across the study period.
